# The effect of phytoestrogens and PAHs on endometriosis and the involvement of gut microbiota, inflammation, and molecular targets

**DOI:** 10.1038/s41598-025-20042-5

**Published:** 2025-10-15

**Authors:** Yanling Chen, Yingtong Jiang, Ziyi Li, Mengyuan Zhu, Amanda Gretta Akimana, Kang Wang, Kun Zhou, Xiaoling Zhang, Xiaoming Ji, Minjian Chen

**Affiliations:** 1 Department of Occupational Medicine and Environmental Health, School of Public Health, Key Laboratory of Public Health Safety and Emergency Prevention and Control Technology of Higher Education Institutions in Jiangsu Province, Nanjing Medical University, Nanjing, 211166 China; 2 State Key Laboratory of Reproductive Medicine, Center for Global Health, School of Public Health, Nanjing Medical University, Nanjing, 211166 China; 3 Key Laboratory of Modern Toxicology of Ministry of Education, School of Public Health, Nanjing Medical University, Nanjing, 211166 China; 4 Department of Epidemiology, Center for Global Health, School of Public Health, Nanjing Medical University, Nanjing, 211166 China; 5 Department of Hygienic Analysis and Detection, School of Public Health, Nanjing Medical University, Nanjing, 211166 China

**Keywords:** Endometriosis, Endocrine disrupting chemicals, Mixed exposure, Inflammatory, Gut microbiota, Reproductive disorders, Public health, Environmental sciences

## Abstract

Endometriosis is a common estrogen-dependent inflammatory disease, yet its complex etiology is not fully understood. Endocrine Disrupting Chemicals (EDCs) exposure disrupts human reproduction, but studies on mixed EDCs and endometriosis risk are Limited. The study Analyzed 2,644 women, assessing 12 phthalates, 8 polycyclic aromatic hydrocarbons, And 6 phytoestrogens in a representative US population. Various statistical models (generalized linear model, partial least squares discriminant analysis, weighted quantile sum, quantile g-computation, restricted cubic spline) were used to explore the link between EDC exposure and endometriosis risk, with mediating effects of lipid metabolism and inflammatory biomarkers examined. Biological mechanisms were identified through an integrated strategy involving target analysis of key chemicals and endometriosis intersections, network establishment, pathway analysis, and target validation. Various statistical models revealed that the gut microbiota metabolite enterolactone (ENL) was negatively associated with endometriosis, while the PAH metabolite 1-Hydroxyphenanthrene (1-OHPHE) was positively associated. Mediation analysis showed that uric acid (UA) and ferritin (Fer) were associated with mediating pathways in the relationships between ENL And decreased risk, And 1-OHPHE and increased risk, respectively. Network and target analysis indicated that ENL affects risk via ESR1, while 1-OHPHE disturbs it through GRB2. ENL can bind to XDH, inhibiting UA production. ENL supplementation may mitigate PAH-induced risks through the PI3K-Akt pathway. In conclusion, higher ENL levels were associated with reduced endometriosis risk, while 1-OHPHE was associated with increased prevalence. Inflammatory mediators UA And Fer demonstrated potential mediating associations in these relationships. ENL levels may be associated with attenuation of 1-OHPHE associations with endometriosis, potentially through gut microbiota-related pathways. These findings emphasize the role of environmental and microbiome interactions in modulating endometriosis risk.

**Supplementary Information:**

The online version contains supplementary material available at 10.1038/s41598-025-20042-5.

## Introduction

Endometriosis is a prevalent And chronic gynecological condition that affects An estimated 5–10% of women of reproductive age^1^. Characterized by the growth of endometrial-like tissue outside the uterus, this condition leads to debilitating symptoms including chronic pelvic pain, infertility, and increased risk of cancer and cardiovascular diseases^2,3^. Although endometriosis is widespread globally, its exact pathogenesis remains poorly understood, and effective preventive measures are still lacking^1^.

Emerging evidence suggests that Endocrine Disrupting Chemicals (EDCs), may play a significant role in endometriosis development^4^. Three classes of EDCs have received particular attention based on epidemiological and mechanistic evidence. Phthalates (PAEs), ubiquitous plasticizers found in consumer products and food packaging^5^Animal and in vitro studies demonstrate their association with endometriosis development through hormonal disruption and inflammatory responses^6,7^. Polycyclic aromatic hydrocarbons (PAHs), combustion byproducts present in air, food, and tobacco^8^show emerging associations with endometriosis risk through mechanisms involving oxidative stress and immune modulation^9,10^. —pathways established as key contributors to endometriosis pathogenesis. Phytoestrogens, plant-derived compounds with estrogenic activity found predominantly in soy products^11^present a complex relationship with endometriosis, as their gut microbiota-mediated metabolism can produce both protective and harmful effects, though their role in endometriosis remains understudied^12–14^.

Critically, real-world exposure to these EDCs occurs through shared pathways, particularly diet, where high-temperature cooking simultaneously generates PAHs while releasing phthalates from food contact materials^5,15^and processed foods containing phytoestrogens often carry phthalates from packaging and PAHs from environmental contamination^16^. However, the key EDCs that cause endometriosis in humans under mixed exposure and the exact mechanisms by which these EDCs influence the pathogenesis of endometriosis remain unclear, and current studies are still limited.

Research on the mechanisms by which mixed exposures to EDCs impact endometriosis in women is crucial for developing effective prevention strategies. Inflammation is a hallmark of endometriosis, with pro-inflammatory cytokines playing a key role in lesion maintenance and pain^17^. Oxidative stress exacerbates this inflammatory response^18^. Additionally, metabolic studies indicate alterations in lipid metabolism in endometriosis, leading to chronic inflammation and oxidative damage, which contribute to lesion persistence and angiogenesis^19^. Lipid metabolism and inflammatory responses are known to mediate the development of various diseases^20,21^. Studies have shown that EDCs not only directly affect hormone levels but may also exacerbate or modulate disease progression by regulating lipid metabolism and inducing inflammatory responses^22^. Based on these findings, we hypothesize that disturbances in inflammation and lipid metabolism may represent key mechanisms through which mixed EDCs exposure influences the development of endometriosis in women.

This cross-sectional study addresses these gaps by examining associations between mixed exposures to PAEs, PAHs, and phytoestrogens and endometriosis risk in a representative U.S. population, with mechanistic focus on inflammatory and lipid metabolic pathways, using advanced statistical approaches to evaluate both individual and joint effects of these EDCs.

## Materials and methods

### Study design and population

**Fig. 1:**
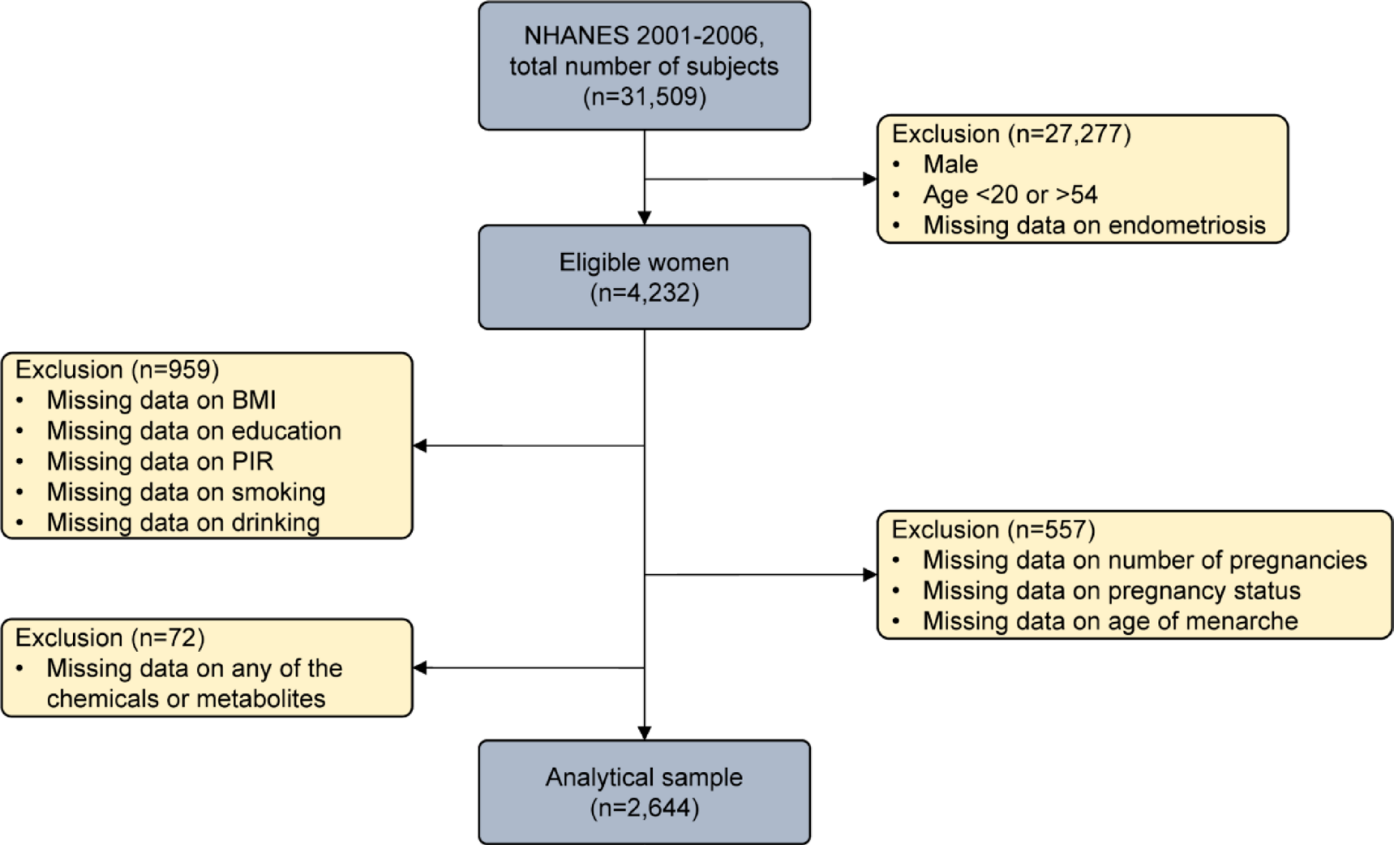
Flow chart of the screening process from NHANES (2001–2006).

This study used data from NHANES (2001–2006), a cross-sectional survey by the National Center for Health Statistics under the CDC, assessing U.S. health and nutrition through interviews and exams. The National Center for Health Statistics (NCHS) is in charge of administering this survey. Research involving Human participants has been conducted in accordance with the Declaration of Helsinki. To participate in NHANES, individuals must complete An informed consent form approved by the NCHS Ethics Review Committee. Using the existing NHANES data for this Analysis does not necessitate further approval from the Institutional Review Board. All research was conducted in accordance with relevant guidelines And regulations. Women aged 20–54 with complete data were included, resulting in a final sample of 2,644 after exclusions (Fig. 1).

## Measurements of chemicals exposure

Urine samples were collected at mobile examination centers, stored at ≤ −20℃, And Analyzed for urinary PAEs, PAHs, And phytoestrogens, which are commonly tested chemical classes with higher exposure levels in women. They were also tested in the same sample population between 2001 And 2006 ^23^. Chemical concentrations were adjusted for urinary creatinine to account for dilution. Analysis of environmental exposures in the NHANES study involved several advanced analytical techniques. Phthalate metabolites were quantified using HPLC-APCI-MS/MS or HPLC-ESI-MS/MS. Urinary PAHs were measured by GC/HRMS following enzymatic hydrolysis, solid-phase extraction, And derivatization, with precise quantification achieved through isotope dilution with 13 C-labeled internal standards. Phytoestrogens were assessed using HPLC-MS/MS from 2001 to 2004, transitioning to HPLC-APPI-MS/MS in 2005–2006. For quality control, a SAS program was used to generate QC charts from exported text files. Final Analyte concentrations were determined using daily calibration curves with duplicate standards, which had typical correlation coefficients greater than 0.99. Abbreviations And detection details are provided in Supplementary Tables 1 And Supplementary Text 1. More details could be found in NHANES website (https://wwwn.cdc.gov/nchs/nhanes/Default.aspx).

## Assessment of endometriosis status

The primary outcome, endometriosis diagnosis, was determined via self-reported responses to the Reproductive Health Questionnaire. Participants who answered “Yes” to “Has a doctor or healthcare professional ever told you that you had endometriosis?” were classified as having endometriosis; “No” indicated no condition. “Don’t know” responses were excluded.

## Covariates

Covariates in this study were grouped into three categories: Demographic, Lifestyle, and Reproductive Health-related. Demographic covariates included age, race, education level, and the poverty income ratio (PIR), with data derived from NHANES demographic information. Race was classified as Hispanic or Non-Hispanic. Education was categorized into four levels: Less than High School, High School, Some College or Associate’s Degree, and College Graduate or Above. PIR was categorized as low income (≤ 1), middle income (> 1 to ≤ 3), and high income (> 3) ^24^. The information was obtained through structured interviews using standardized questionnaires, which were conducted by trained NHANES personnel.

Lifestyle covariates included smoking status, alcohol consumption, and body mass index (BMI). Smoking status was categorized into three groups: non-smokers, current smokers, and former smokers, based on responses to the questions, “Have you smoked at least 100 cigarettes in your lifetime?” and “Do you currently smoke?“. Alcohol consumption was classified as either non-drinker or drinker. BMI was categorized into four groups: underweight (BMI < 18.5 kg/m²), normal weight (18.5 kg/m² ≤ BMI < 25 kg/m²), overweight (25 kg/m² ≤ BMI < 30 kg/m²), and obese (BMI ≥ 30 kg/m²). These data were obtained from physical measurements and questionnaire responses. BMI data were collected through body measurements using standardized equipment in mobile examination centers^25^. Meanwhile, information regarding smoking and drinking status was gathered via questionnaire responses.

Reproductive health-related covariates included pregnancy status (as determined by pregnancy test results), age of menarche (the age at the first menstrual period), and gravidity (the number of pregnancies). Pregnancy status was determined through laboratory urine tests conducted in MECs. Age of menarche and gravidity were collected through interviewer-administered reproductive health questionnaires by trained personnel.

## Lipid metabolism and inflammatory biomarkers measurement

Biomarkers included lipid metabolism indicators such as triglycerides (TG), low-density lipoprotein cholesterol (LDL-C), high-density lipoprotein cholesterol (HDL-C), and total cholesterol (TC), as well as inflammatory markers including uric acid (UA), ferritin (Fer), and C-reactive protein (CRP). These were selected based on literature and assessed in NHANES^26^. HDL-C, TG, And TC were Analyzed using the Hitachi 704 Analyzer, Hitachi 717 And Hitachi 912 Analyzers. LDL-C was calculated from the measured values of TC, TG, And LDL-C using the following formula, applicable for the period from 2001 to 2006^27^:

$$\:\mathbf{L}\mathbf{D}\mathbf{L}-\mathbf{C}\:=\:\mathbf{T}\mathbf{C}\:-\:\mathbf{H}\mathbf{D}\mathbf{L}-\mathbf{C}\:-\:(\mathbf{T}\mathbf{G}/5)$$

Fer was measured using the Bio-Rad Laboratories assay from 2001 to 2003, And with the Hitachi 912 from 2004 to 2006. CRP levels were quantified by nephelometry from 2001 to 2006. UA levels were measured using the Beckman Synchron LX20 from 2001 to 2006^28–30^

### Key chemicals (ENL, 1-OHPHE) and endometriosis targets prediction

To gather detailed information on the characterization and physicochemical properties of key chemicals, the PubChem database (https://pubchem.ncbi.nlm.nih.gov/) was searched using chemical identifiers CAS: 185254-87-9, 2433-56-9. Target prediction was carried out using the SuperPred and SwissTarget platforms, where Canonical SMILES notation was used for input. The resulting predicted targets were mapped to corresponding gene names via Uniprot (https://www.uniprot.org/). These gene targets were associated with the ENL And 1-OHPHE. Additionally, gene targets specifically related to endometriosis were identified through the GeneCards database (https://www.genecards.org/).

## Construction of key chemicals and endometriosis intersection targets network

Protein-protein interaction (PPI) networks for the identified intersection targets were constructed using the STRING database (https://cn.string-db.org/). The interaction data were downloaded in TSV format And imported into Cytoscape 3.10.2 for network visualization and further analysis.

## GO and KEGG enrichment analyses

Gene Ontology (GO) enrichment and Kyoto Encyclopedia of Genes and Genomes (KEGG) pathway Analyses were conducted for the target genes using R. The selection of enriched terms was based on the clustering results provided by R. We sorted the pathways by the FDR-corrected p-values And selected the top 20 pathways with FDR < 0.05 ^31^. The analysis was carried out using the clusterProfiler package in R.

### Molecular Docking

Molecular docking simulations were performed using AutoDock Vina 1.1.2. The SDF files for the key chemicals (ENL And 1-OHPHE) were obtained from PubChem (https://pubchem.ncbi.nlm.nih.gov/), while the PDB files for Human Estrogen receptors 1 (ESR1), Xanthine Dehydrogenase (XDH), And Growth factor receptor-bound protein 2 (GRB2) (PDB codes: 1a52, 2ckj, 1cj1) were retrieved from the Protein Data Bank (https://www.rcsb.org/). The binding affinities of Enterolactone (ENL) And 1-Hydroxyphenanthrene (1-OHPHE) to the target proteins were evaluated based on their binding energy values. The docking simulation results were visualized using PyMOL (version 1.2) and Discovery Studio (version 2.4). Strong binding potential was defined as docking energy ≤ −7.0 kcal/mol^32^.

### Molecular dynamics simulation

The molecular docking system was further refined using Gromacs 2023. A solvated system under periodic boundary conditions, ensuring appropriate size and electroneutrality, was prepared for subsequent molecular dynamics simulations^33,34^. The molecular dynamics simulations were carried out in several stages: energy minimization^35^equilibration in the isothermal-isotropic ensemble^36,37^equilibration in the isothermal and isobaric ensemble^38^and production stage simulation over 100 ns.

### Statistical analysis

To characterize the study population, we calculated the mean and standard deviation (SD) for continuous variables, and the count and percentage for categorical variables. To achieve a normal distribution, the concentrations of the selected urinary creatinine adjusted EDCs and biomarkers were log-transformed. Sample weights were applied to account for the complex, multistage sampling design of NHANES^39^. Logistic regression models were used to examine the associations between individual EDCs and the risk of endometriosis. Odds ratios (ORs) with corresponding 95% confidence intervals (CIs) were reported.

Partial least squares discriminant analysis (PLS-DA) was conducted to assess the effects for confirmatory purpose. PLS-DA was conducted as an exploratory tool to identify exposure patterns associated with endometriosis, with covariates included as additional variables in the model to account for potential confounding factors. This approach allows for simultaneous consideration of multiple EDCs and confounding variables in pattern recognition. Consistent covariate adjustments were applied across all analyses. Significant associations were identified by integrating the results from PLS-DA with Variable Importance in Projection (VIP) > 1 and logistic regression (*P* < 0.05)^40^.R packages mixOmics was adopted to perform the analysis.

To further evaluate the combined impact of mixed EDCs, we employed weighted quantile sum (WQS), quantile g-computation. The WQS regression analysis were used to examine both the overall and individual effects of EDCs on the risk of endometriosis. In this approach, chemicals were weighted according to their quantiles, and a weighted index was used as a single exposure term in a logistic regression model. The dataset was divided into two subsets: training (40%) and validation (60%). The WQS index for each participant was calculated by averaging the weights of each EDC. The relationship between endometriosis risk and the WQS index was then assessed in the validation subset^41^. Consistent covariate adjustments were applied to maintain analytical consistency. R package gWQS was adopted to perform the analysis.

The quantile g-computation model was employed to examine both the overall and individual effects of EDCs on the risk of endometriosis. This model is a parameterized generalized linear model built upon quantile g-computation, designed to assess the effect of simultaneously increasing all exposures in the mixture by one quantile^42^. In cases where an EDC had effects in both directions, the weight indicated the proportion of the exposure’s influence on endometriosis risk. The relationship between each individual EDC endpoint and the mixture of EDCs was examined separately. The resulting models provided scaled effect sizes, variable-specific coefficients, and overall model fit P-values. The analysis was carried out using the qgcomp package in R.

To further evaluate the and potential non-linear relationships on the risk of endometriosis, restricted cubic spline (RCS) were applied to explore potential non-linear relationships between EDCs And endometriosis risk, with curves plotted to visualize these associations. Subgroup Analyses were performed to assess whether the associations between key EDCs And endometriosis risk varied by covariates. Age and age at menarche were categorized with cutoff points at 35 years And 13 years, respectively, to evaluate the effect of aging on endometriosis risk^43^. R package rms was adopted to perform the Analysis. All the Analyses were performed in R version 4.3.2.

## Results

### Study population baseline characteristics

Table 1 presents the survey-weighted descriptive statistics of the study population. The endometriosis group was significantly older on average (41.04 vs. 39.21 years, *P* = 0.001) and had a higher proportion of non-Hispanic individuals (94.99% vs. 87.35%, *P* < 0.001). In terms of education, a greater percentage of endometriosis patients had completed high school or obtained a GED or equivalent (32.93% vs. 22.92%, *P* = 0.019). We acknowledge that there is a statistically significant age difference between the two groups (normal female group: 39.21 ± 9.32 years vs. endometriosis group: 41.04 ± 7.62 years, *P* = 0.001). Although the absolute age difference is relatively small (1.83 years), considering that age may potentially have an impact on the biological indicators of our study, we adjusted age as a covariate in all subsequent statistical analyses.

**Table 1:** Population characteristics of individuals in NHANES 2001–2006 (*n* = 2,644).

		Normal female	Endometriosis female	*P*
Age (mean (SD))		39.21 (9.32)	41.04 (7.62)	**0.001**
Race (%)				**< 0.001**
	Non-Hispanic	87.35	94.99	
	Hispanic	12.65	5.01	
BMI (%)				0.998
	Under weight	2.38	2.42	
	Normal weight	36.46	36.92	
	Over weight	26.69	26.26	
	Obesity	34.47	34.39	
Smoker (%)				0.071
	Never smoker	50.62	43.01	
	Former smoker	21.6	21.62	
	Current smoker	27.78	35.37	
Alcohol (%)				0.487
	No	17.54	19.45	
	Yes	82.46	80.55	
Education (%)				**0.019**
	High school and below	15.37	9.32	
	High school graduate/GED or equivalent	22.92	32.93	
	Some college or AA degree	36.64	34.76	
	College graduate or above	25.07	22.99	
PIR (%)				
	PIR ≤ 1	14.37	13.6	0.775
	PIR 1–3	33.57	31.11	
	PIR > 3	52.06	55.29	
Age of menarche (mean (SD))		12.63 (1.61)	12.39 (1.76)	0.140
Number of pregnancies (mean (SD))		3.00 (2.001)	2.81 (1.51)	0.089
Pregnancy (%)				0.051
	No	93.36	97.19	
	Yes	6.64	2.81	

### Exposure levels of paes, pahs, and phytoestrogens and endogenous metabolites levels

Supplementary Table 1 presents the concentrations of PAEs, PAHs, And phytoestrogens. PAEs concentrations ranged from 0.90 ng/ml (MMP) to 139.39 ng/ml (MEP). PAHs levels varied widely, with 2-OHPHE at 55.86 ng/ml And 2-OHNAP at 3316.89 ng/ml. Among phytoestrogens, ENL had the highest median concentration at 370.14 ng/ml. Detailed levels of various metabolites are provided in Supplementary Table 1.

### Logistic regression and PLS-DA analysis of the association between selected chemicals and endometriosis risk

**Fig. 2:**
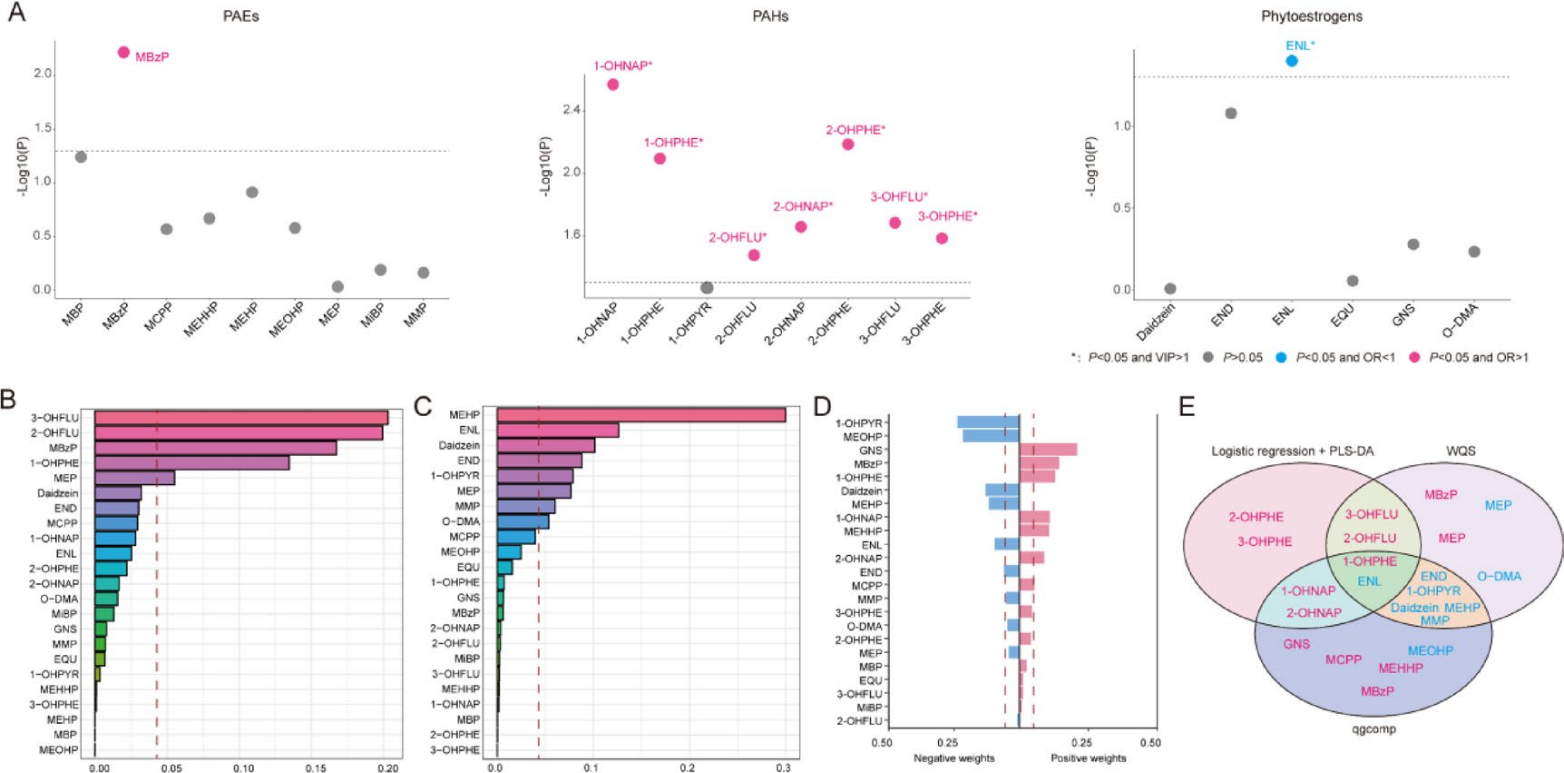
Associations between EDCs exposure and endometriosis risk. **(A)** Association analysis of EDCs exposure and endometriosis risks: Logistic regression and PLS-DA. **(B)** Positive WQS regression weights between EDCs exposure and endometriosis risks; **(C)** Negative WQS regression weights between EDCs exposure and endometriosis risks. **(D)** Quantile g-computation model regression index weights of the mixture on endometriosis risk. **(E)** Key EDCs associated with endometriosis identified through various statistical model. *PAEs* Phthalates *PAHs* Polycyclic aromatic hydrocarbons *PLS-DA* partial least squares discriminant analysis *WQS* weighted quantile sum *1-OHPHE* 1-Hydroxyphenanthrene *ENL* enterolactone.

Logistic regression revealed significant associations between specific chemicals and endometriosis. ENL (OR: 0.828, 95% CI: 0.691, 0.991, *P* = 0.040) was found to be significantly negatively correlated with the presence of endometriosis, while seven PAHs, 1-OHNAP (OR: 1.238, 95% CI: 1.082, 1.418, *P* = 0.003), 2-OHNAP (OR: 1.409, 95% CI: 1.053, 1.884, *P* = 0.022), 3-OHFLU (OR: 1.352, 95% CI: 1.050, 1.742, *P* = 0.021), 2-OHFLU (OR: 1.407, 95% CI: 1.029, 1.924, *P* = 0.033), 3-OHPHE (OR: 1.426, 95% CI: 1.045, 1.944, *P* = 0.026), 1-OHPHE (OR: 1.679, 95% CI: 1.153, 2.446, *P* = 0.008) And 2-OHPHE (OR: 1.529, 95% CI: 1.133, 2.062, *P* = 0.006), along with MBzP (OR: 1.354, 95% CI: 1.096, 1.673, *P* = 0.006), were positively associated with the condition (Fig. 2A, Supplementary Table 2).

PLS-DA further validated the results from logistic regression (except for MBzP) and confirmed the potential association between identified PAHs and phytoestrogens with endometriosis (Fig. 2A, Supplementary Table 2).

### Association of chemicals co-exposure with endometriosis risk

The WQS model was used to estimate the combined effect of mixed EDC exposures on the risk of endometriosis. In the model, it is important to note that in the negative direction, O-DMA, MMP, MEP, 1-OHPYR, END, Daidzein, ENL and MEHP exhibited a higher Weight, while MEP, 1-OHPHE, MBzP, 2-OHFLU And 3-OHFLU showed a higher weight in the positive direction (Fig. 2B-C).

In quantile g-computation model, GNS, MBzP, 1-OHPHE, 1-OHNAP, MEHHP, 2-OHNAP, And MCPP were identified as positive contributors to the quantile g-computation scores, while 1-OHPYR, MEOHP, Daidzein, MEHP, ENL, END, and MMP were identified as negative contributors to the quantile g-computation scores in estimating the endometriosis risk for each EDC (Fig. 2D). The Venn diagram demonstrated that the combination of logistic regression with PLS-DA, WQS, And quantile g-computation consistently identified 1-OHPHE and ENL, a gut microbiota-derived metabolite from phytoestrogens, as the key exposures associated with endometriosis (Fig. 2E).

### Logistic regression and PLS-DA model analysis of the association between inflammatory and lipid biomarkers and endometriosis risk

**Fig. 3:**
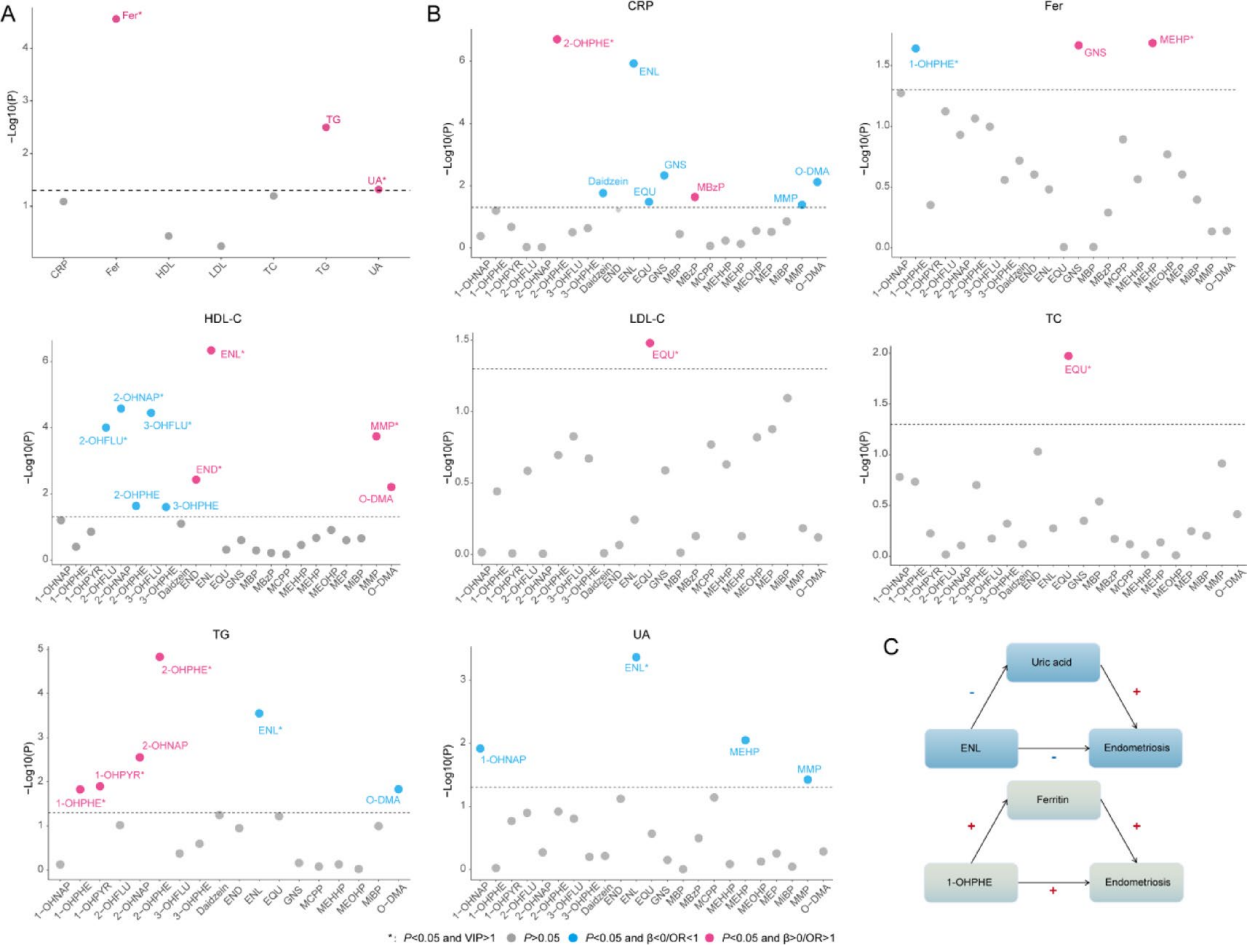
Associations between biomarkers, EDCs exposure, and endometriosis risk: Statistical analyses and mediation associations. **(A)** Association analysis of lipid metabolism, inflammatory biomarkers and endometriosis: Logistic regression and PLS-DA. **(B)** Association analysis of EDCs and lipid metabolism, inflammatory biomarkers: Linear regression and PLS-DA. **(C)** Mediation analysis of the associations between of PAHs and phytoestrogens on the risk of endometriosis. *CRP* C-reactive protein *Fer* ferritin *HDL-C* high-density lipoprotein cholesterol *LDL-C* low-density lipoprotein cholesterol *TC* total cholesterol *TG* triglycerides *UA* uric acid *1-OHPHE* 1-Hydroxyphenanthrene *ENL* enterolactone.

We examined the relationship between inflammatory markers (CRP, Fer, UA) and lipid biomarkers (HDL-C, LDL-C, TC, TG) with endometriosis risk. Logistic regression analysis indicated that higher Fer levels significantly associated with increased the risk of endometriosis (OR: 1.599, 95% CI: 1.306, 1.957, *P* < 0.001), as did TG (OR: 1.880, 95% CI: 1.251, 2.826, *P* = 0.003) and UA (OR: 1.803, 95% CI: 1.005, 3.232, *P* = 0.048) (Fig. 3A, Supplementary Table 3). In the PLS-DA model, Fer and UA showed positive correlations with endometriosis risk, while TG was not significantly associated (Fig. 3A, Supplementary Table 3).

### Linear regression and PLS-DA model analysis of the association between EDCs and inflammatory and lipid biomarkers

As shown in Supplementary Table 4, Linear regression analysis revealed several significant associations between EDCs and Lipid And inflammatory biomarkers. HDL-C was positively correlated with MMP, O-DMA, ENL And END, but negatively correlated with 2-OHFLU, 2-OHNAP, 2-OHPHE, 3-OHFLU And 3-OHPHE. EQU was positively correlated with TC And LDL-C. TG showed a negative correlation with O-DMA And ENL, And a positive correlation with 2-OHPHE, 2-OHNAP, 1-OHPHE, And 1-OHPYR. Fer was negatively correlated with MEHP And GNS, but positively correlated with 1-OHPHE. CRP was positively correlated with 2-OHPHE And MBzP, And negatively correlated with MMP, Daidzein, O-DMA, ENL, EQU, And GNS. UA was negatively correlated with MEHP, MMP, ENL, and 1-OHNAP (Fig. 3B).

In the PLS-DA model, HDL-C remained significantly correlated with 2-OHFLU, 2-OHNAP, 3-OHFLU, MMP, ENL And END; EQU remained significantly correlated with TC And LDL-C; TG remained significantly correlated with ENL, 1-OHPHE, 2-OHPHE And 1-OHPYR; Fer remained significantly correlated with MEHP And 1-OHPHE; CRP remained significantly correlated with 2-OHPHE and ENL; and UA remained significantly correlated with ENL (Fig. 3B).

### Association analysis of potential mediating pathways

Based on the results from Sect. 3.3 And 3.5 to 3.6, we explored potential mediating pathways through which inflammatory factors may link phytoestrogens and PAHs exposures with endometriosis. We conducted pairwise regression analyses to examine the associations between: (1) EDCs and inflammatory biomarkers (linear regression), and (2) EDCs/inflammatory biomarkers and endometriosis (logistic regression).

These Analyses revealed potential indirect pathways consistent with mediation. Specifically, ENL showed inverse associations with both UA levels And endometriosis prevalence, while UA was positively associated with endometriosis, suggesting a potential pathway where ENL may be associated with reduced endometriosis prevalence through its association with lower UA levels. Similarly, 1-OHPHE demonstrated positive associations with both Fer levels and endometriosis prevalence, while elevated Fer was associated with increased endometriosis prevalence, indicating a potential pathway Linking 1-OHPHE exposure to endometriosis through inflammatory processes (Fig. 3C).

### Dose-response relationships between urine EDCs with endometriosis

**Fig. 4:**
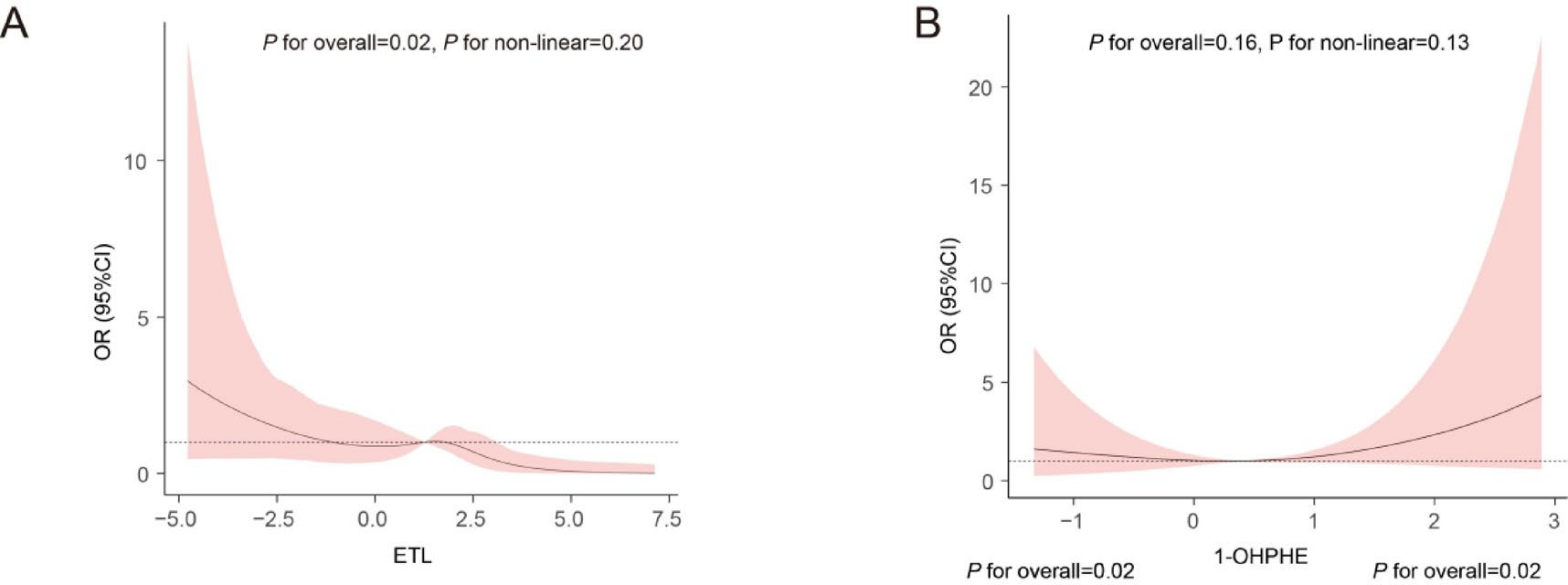
Dose-response And subgroup Analysis of ENL And 1-OHPHE exposure on endometriosis risk. **(A-B)** Dose-response relationships between log-transformed urinary ENL (**A**) And 1-OHPHE (**B**) levels And endometriosis risk, with the shaded area representing the 95% confidence interval. *1-OHPHE* 1-Hydroxyphenanthrene *ENL* enterolactone *OR* Odds ratios *CI* confidence intervals.

Building on the significant findings from the logistic regression and PLS-DA analysis, we further investigated the relationship between log-transformed urine levels of significant chemical substances and endometriosis risk using RCS (Fig. 4A-B).

### Subgroup analysis

Subgroup Analyses were conducted to examine the influence of various covariates on the relationship between urinary ENL, 1-OHPHE, and endometriosis risk (Supplementary Tables 5–6). Based on the results from both the logistic regression And PLS-DA models, we found that urinary ENL levels were significantly negatively correlated with endometriosis risk in participants who were obese, not pregnant, And had fewer than three pregnancies. For 1-OHPHE, a significant positive correlation with endometriosis risk was observed in individuals who were under 35 years old, non-Hispanic, consumed alcohol, had lower educational attainment, had lower socioeconomic status, and had either been pregnant or not pregnant, as well as those with three or more pregnancies. Additional subgroup analyses are presented in Supplementary Fig. S1.

### Identification of common targets and PPI network construction

**Fig. 5:**
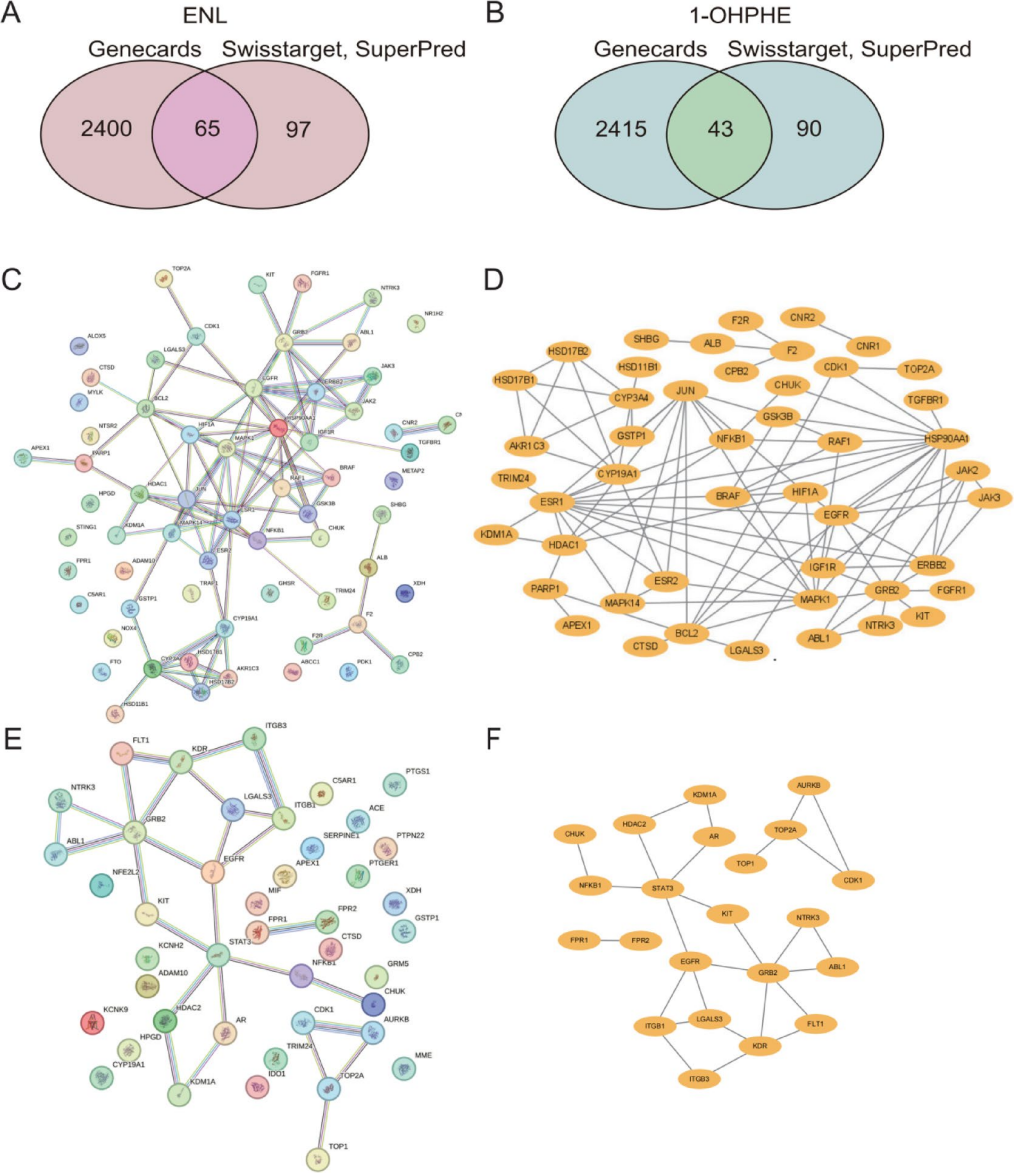
Identification of common targets And interaction networks between ENL, 1-OHPHE, and endometriosis. **(A)** Venn diagram of common targets between ENL and endometriosis. **(B)** Venn diagram of common targets between 1-OHPHE and endometriosis. **(C-D)** PPI network for ENL and endometriosis targets. **(E-F)** PPI network for 1-OHPHE and endometriosis targets. *1-OHPHE* 1-Hydroxyphenanthrene *ENL* enterolactone.

To explore the underlying mechanisms of ENL in endometriosis, we predicted potential target genes for both ENL And endometriosis using SuperPred, SwissTargetPrediction, And GeneCards. A Venn diagram, generated by intersecting the ENL targets with 2,465 endometriosis-related protein coding targets, revealed 65 common targets (Fig. 5A). Subsequently, a subsequent PPI network was constructed using STRING And visualized with Cytoscape 3.10.2. After excluding isolated targets, the final PPI network highlighted several critical interactions (Fig. 5C-D), with ESR1 showing the highest degree centrality. Notably, XDH, considered a key player in the purine metabolism pathway, particularly in UA production, is also present in this PPI network. For PAHs, target prediction for 1-OHPHE identified 133 targets using SuperPred And SwissTargetPrediction. After cross-referencing with GeneCards, 43 targets were selected (Fig. 5B). PPI network analysis revealed GRB2 as the most prominent target, exhibiting the highest degree value (Fig. 5E-F).

#### GO and KEGG enrichment analyses

To explore the potential biological pathways And functions of ENL And 1-OHPHE in endometriosis, we performed GO And KEGG enrichment Analyses using the R package clusterProfiler on 65 common targets for ENL And 43 common targets for 1-OHPHE (Fig. 5A).

**Fig. 6:**
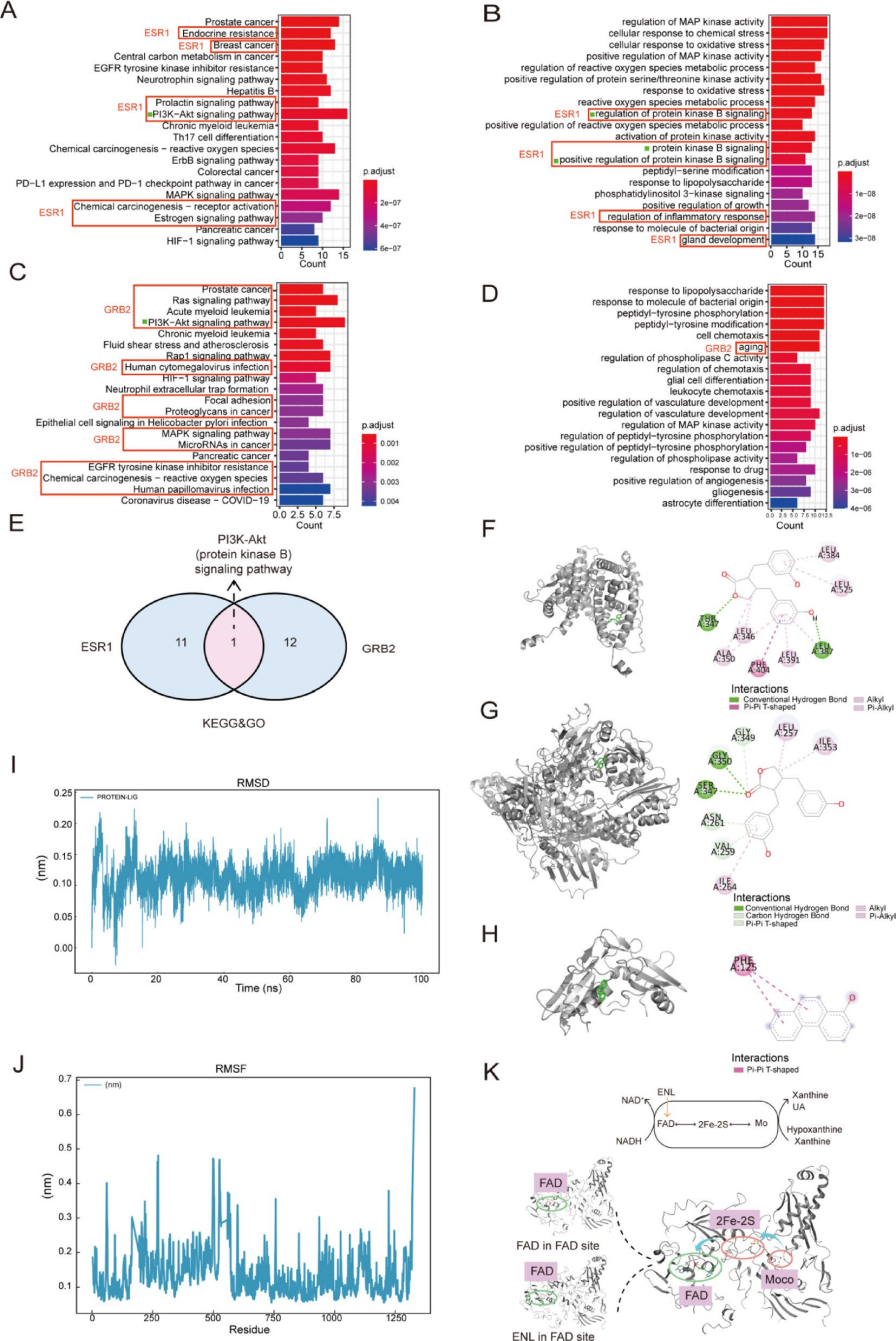
Target identification And molecular interactions of ENL And 1-OHPHE in endometriosis. **(A)** KEGG enrichment analysis of biological processes for ENL targets in endometriosis. **(B)** GO pathway analysis of ENL targets in endometriosis. **(C)** KEGG enrichment Analysis of biological processes for 1-OHPHE targets in endometriosis. **(D)** GO pathway Analysis of 1-OHPHE targets in endometriosis. **(E)** Intersection of KEGG and GO enrichment results for ESR1 and GRB2. **(F-G)** Molecular docking analysis of ENL binding to ESR1 and XDH complexes. **(H)** Molecular docking Analysis of 1-OHPHE binding to GRB2 complexes. **(I)** RMSD from molecular dynamics simulations of XDH and ENL. **(J)** RMSF from molecular dynamics simulations of XDH with ENL. The red boxes highlight the pathways among the top 20 enriched pathways that involve ESR1 and GRB2, while the green squares indicate the pathways that are shared by both. **(K)** Overview of XDH catalytic mechanism in oxidizing hypoxanthine to xanthine, and xanthine to UA via Moco, [2Fe-2 S], and FAD. ENL blocks the electron transfer of Moco, [2Fe-2 S], and FAD in the enzymatic process by binding to the FAD site. *RMSD* root mean square deviation *RMSF* root mean square fluctuation *ENL* enterolactone *UA* uric acid *FAD* Flavin adenine dinucleotide *Moco* Molybdenum Cofactor.

KEGG pathway analysis for ENL targets (Fig. 6A) revealed enrichment in pathways such as prostate cancer, endocrine resistance, breast cancer, central carbon metabolism in cancer, and EGFR tyrosine kinase inhibitor resistance. GO enrichment analysis (Fig. 6B) identified several key biological processes, including regulation of MAPK activity, cellular response to chemical stress, cellular response to oxidative stress, positive regulation of MAPK activity, and regulation of reactive oxygen species metabolism.

For 1-OHPHE targets, KEGG pathway analysis (Fig. 6C) highlighted significant pathways such as prostate cancer, Ras signaling pathway, acute myeloid leukemia, PI3K-Akt signaling pathway, and chronic myeloid leukemia. GO enrichment analysis (Fig. 6D) revealed enriched processes primarily related to response to lipopolysaccharide, response to molecules of bacterial origin, peptidyl-tyrosine phosphorylation, peptidyl-tyrosine modification, and cell chemotaxis.

For ESR1, it was found to be involved in the top 20 KEGG pathways, including endocrine resistance, breast cancer, prolactin signaling pathway, PI3K-Akt signaling pathway, And chemical carcinogenesis-receptor activation. The top 20 GO pathways for ESR1 included regulation of protein kinase B, kinase B signaling, positive regulation of kinase B signaling, regulation of inflammatory response, and gland development. GRB2, meanwhile, was involved in the top 20 KEGG pathways, such as prostate cancer, Ras signaling pathway, acute myeloid leukemia, PI3K-Akt signaling pathway, Human cytomegalovirus infection, focal adhesion, proteoglycans in cancer, MAPK signaling pathway, microRNAs in cancer, EGFR tyrosine kinase inhibitor resistance, chemical carcinogenesis-reactive oxygen species, And Human papillomavirus infection. Additionally, aging was enriched in the top 20 GO pathways for GRB2. We found that both ESR1 and GBR2 shared the PI3K-Akt signaling pathway (Fig. 6E).

### Molecular Docking Analysis of ENL And 1-OHPHE binding to key targets in endometriosis

To explore the potential binding interactions of ENL with key target proteins identified through network analysis, molecular docking simulations were performed with ESR1 and XDH. The results, shown in Fig. 6F-G, revealed binding energies of −9.8 kcal/mol for the ENL-ESR1 complex and − 8.1 kcal/mol for the ENL-XDH complex, indicating strong binding affinities of ENL for both proteins. Specifically, ENL interacted with ESR1 by forming conventional hydrogen bonds with THR347 and LEU387, π-π T-shaped interactions with PHE404, alkyl interactions with LEU346 and ALA350, and π-alkyl interactions with LEU346, ALA350, LEU391, LEU525, and LEU384. In the ENL-XDH complex, ENL formed conventional hydrogen bonds with SER347 and GLY350, a carbon-hydrogen bond with GLY349, and π-donor hydrogen bonds with ASN261 and VAL259 (Fig. 6G), Therefore, the electron transfer of Molybdenum Cofactor (Moco), [2Fe-2 S] and Flavin adenine dinucleotide (FAD) in the process of enzyme catalysis for UA production was blocked^44^. Additionally, ENL established alkyl interactions with LEU257 and ILE353, and π-alkyl interactions with ILE264. These findings suggest that ENL binds effectively to both ESR1 and XDH, supporting the hypothesis that ENL may mitigate endometriosis by modulating ESR1 activity and inhibiting XDH-mediated UA production.

To further investigate the binding interactions of 1-OHPHE with the key target GRB2, additional molecular docking simulations were conducted. As shown in Fig. 6H, the binding energy for the 1-OHPHE-GRB2 complex was − 6.1 kcal/mol, indicating stable binding affinity. Specifically, 1-OHPHE formed π-π T-shaped interactions with PHE125.

#### Molecular dynamics simulation of the ENL-XDH complex

As mediation analysis revealed that UA mediates the relationship between ENL and decreased risk (Fig. 3C), in the above targets, considering that XDH is An upstream target that directly produces UA, we selected the ENL-XDH complex for molecular dynamics simulation within 100 ns under simulated water environment. The root mean square deviation (RMSD) of the protein-ligand complex during the simulation (Fig. 6I) remained stable at approximately 0.1 nm, indicating that the ENL-XDH complex maintained a consistent and stable conformation throughout the simulation period. Additionally, the root mean square fluctuation (RMSF) analysis revealed that residues near the active site of XDH showed reduced fluctuation and stabilized after the binding of ENL to the enzyme (Fig. 6J). These findings suggest that ENL binding may influence the conformation of the active site, potentially modulating the enzymatic function of XDH by occupying the FAD site in the normal catalytic process (Fig. 6K).

### The positive association between PAH exposure and endometriosis appears weaker in individuals with higher ENL levels

Given ENL’s potential protective role, we further investigated its impact on PAHs exposure. To explore potential intervention strategies for PAHs-related endometriosis risk, we analyzed the effect of serum ENL levels (categorized by median) on the relationship between PAHs And endometriosis risk. Supplementary Table 7 shows that, for individuals with low ENL levels, PAHs such as 1-OHNAP, 1-OHPHE, 2-OHFLU, 2-OHNAP, 2-OHPHE, 3-OHFLU, 3-OHPHE were positively associated with endometriosis risk. In contrast, for individuals with high ENL levels, these positive associations were no longer observed, suggesting that ENL may mitigate the harmful effects of PAHs on endometriosis risk. Since ENL is a ligand-derived metabolite produced by the gut microbiota, the results suggest that the gut microbiota play a role in this protective effect.

To investigate the underlying mechanisms, we examined the shared targets of ENL And 1-OHPHE and found that ESR1 (target of ENL) and GRB2 (target of 1-OHPHE) both co-participate in the PI3K-Akt (kinase B) signaling pathway. The activation of PI3K-Akt signaling is promoted by ESR1 translocation from the nucleus to the plasma membrane, while GRB2 facilitates PI3K recruitment and activation via its interaction with Gab1, thereby initiating the PI3K-Akt pathway and regulating cell survival and proliferation^45–47^. This suggested that 1-OHPHE and ENL exerted their potential role by interfering with PI3K-Akt signaling pathway.

## Discussion

In this cross-sectional study of American women, the results indicated that the phytoestrogen ENL was negatively associated with endometriosis risk, whereas the PAH metabolite 1-OHPHE was consistently positively associated with the risk under mixed exposure. The subgroup Analysis identified the potentially susceptible populations. Mediation Analysis suggests that UA And Fer may mediate the association of ENL and 1-OHPHE with endometriosis, respectively. Molecular docking analysis indicated that ENL may reduce the risk of endometriosis by binding to ESR1 And XDH. In contrast, 1-OHPHE may increase the risk by interacting with GRB2. Through molecular dynamics simulation of XDH, a direct catalytic target of UA, we found that ENL may inhibit UA production by occupying the FAD site of XDH. The supplementation with ENL may represent a viable strategy to mitigate the adverse effects of certain PAHs exposure on the risk of endometriosis.

Phytoestrogens refer to a class of plant-derived compounds that exhibit estrogen-like effects. They have been widely studied for their ability to interact with estrogen receptors, particularly for their potential therapeutic role in endometriosis. Previous studies suggest that phytoestrogens may help manage endometriosis symptoms by regulating estrogen levels, inhibiting cell proliferation, modulating hormone signaling pathways, and controlling inflammation^48^. Additionally, epidemiological studies have found that phytoestrogens may offer protective effects against the development of endometriosis, as they are closely related to the regulation of hormone levels, immune markers, and inflammatory indicators, showing a negative correlation with endometriosis risk^49^. However, the involvement of specific phytoestrogen metabolites in endometriosis among humans under mixed exposure remains unknown. Notably, phytoestrogen metabolites like ENL and END have been identified as phytoestrogens with weak estrogenic activity, as well as anti-estrogenic properties. Studies indicate that these anti-estrogenic effects may contribute to breast cancer prevention^50^. Our research also suggests that phytoestrogen ENL may have potential in reducing the risk of endometriosis. Phytoestrogens typically enter the body in their native form, but their bioactivity is largely dependent on the metabolic actions of the gut microbiota. The gut microbiota can convert phytoestrogens into various active metabolites, thereby influencing their physiological effects on the host^51^. The composition and diversity of the gut microbiota directly impact the metabolism of phytoestrogens and their biological effects. Individual differences in gut microbiota may lead to variability in the biological effects of phytoestrogens, as different microbiota may metabolize phytoestrogens differently^52^. ENL is a metabolite formed from lignans through the action of gut microbiota^53^. Dysbiosis in the gut microbiota may reduce the metabolic efficiency of lignans, thereby affecting the production and bioavailability of ENL. Therefore, the health status and diversity of the gut microbiota are critical for the proper biological function of ENL, directly influencing its generation and biological effects.

In contrast to the potential protective effects of phytoestrogens, PAHs, known endocrine disruptors, are positively correlated with the risk of endometriosis. PAHs are commonly found in air pollution, cigarette smoke, and charred food, and they can mimic or block the action of natural hormones, disrupting the endocrine system. This can lead to hormone imbalance, promote abnormal tissue growth, and trigger inflammatory responses, thereby increasing the risk of endometriosis. One study found that the accumulation levels of PAHs in the abdominal adipose tissue of patients with endometriosis were significantly higher^54^. PAHs may exacerbate the risk of endometriosis by promoting chronic inflammation, oxidative stress, and hormone imbalance^55^.

This study found a negative correlation between ENL And UA, And a positive correlation between 1-OHPHE and Fer. Moreover, both UA and Fer levels were positively correlated with the risk of endometriosis. A previous study showed that phytoestrogens could reduce UA production^56^and experimental studies demonstrated that exposure to PAHs in rainbow trout increased Fer levels^57^. Previous research also indicated that elevated levels of Fer and UA might serve as risk biomarkers for endometriosis^58,59^. Research has shown that the expression of Fer confers resistance to apoptosis in endometriosis cells^60^.

**Fig. 7:**
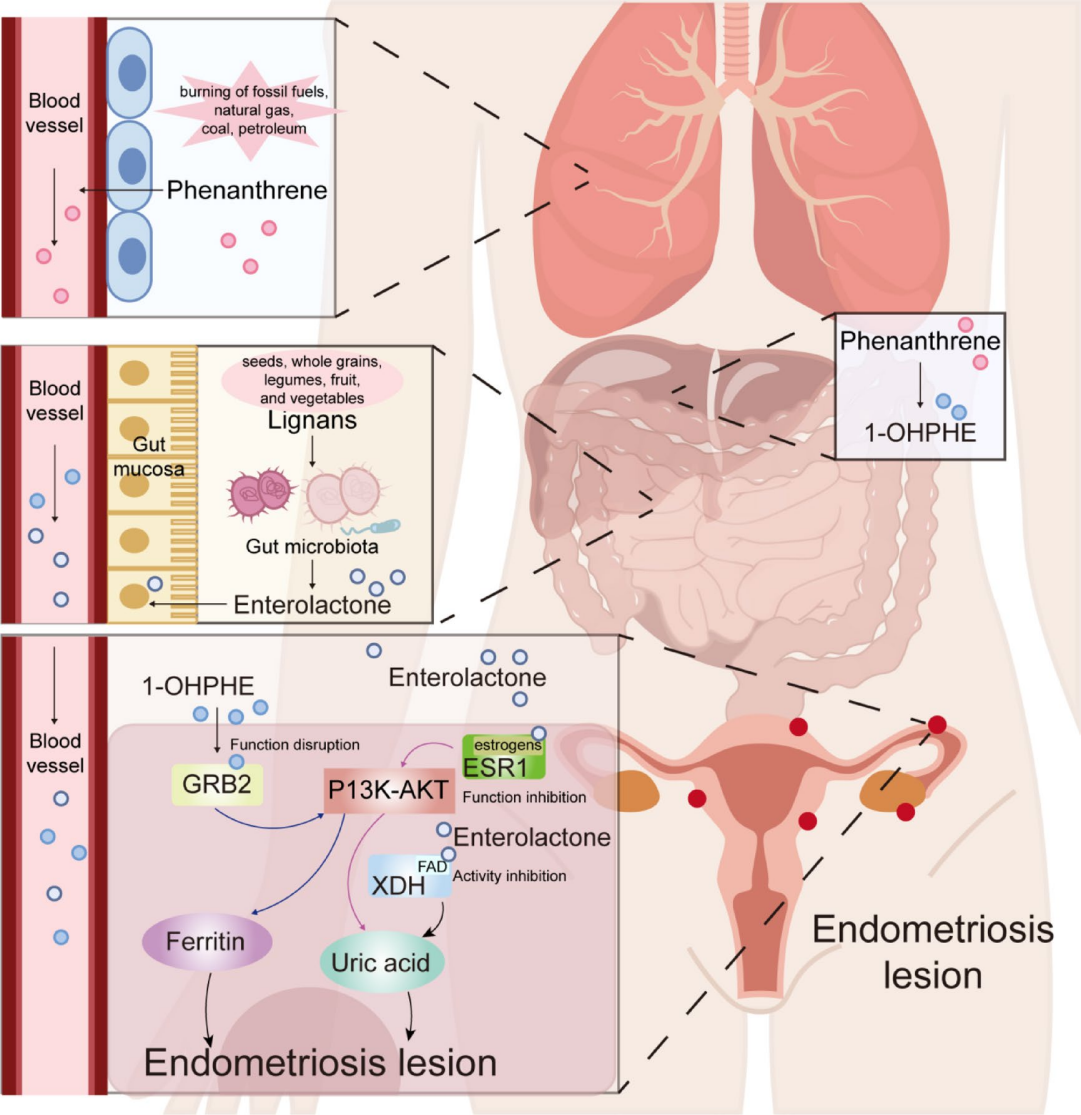
Mechanisms underlying the role of enterolactone and 1-hydroxyphenanthrene in endometriosis development. *1-OHPHE* 1-Hydroxyphenanthrene.

Additionally, the construction of 1-OHPHE, ENL and endometriosis targets network provided insights into the potential protective mechanisms of phytoestrogens in endometriosis. We found that ENL can bind stably to the FAD active site of XDH, which plays a key role in electron transfer for UA production, suggesting that ENL may reduce UA levels and alleviate inflammation by inhibiting XDH activity, thereby reducing the risk of endometriosis. ENL also binds to the ESR1 receptor, disrupting its normal function. Estrogen regulates the growth, proliferation, and shedding of the endometrial tissue by binding to the ESR1 receptor^61^. ESR1 promotes the proliferation of endometrial epithelial cells through PI3K-Akt pathways, a process that has been validated in mouse models^62^. Furthermore, studies indicate that compared to the control group, ESR1 levels in the secretory-phase endometrium of women with endometriosis are significantly elevated^63^. Therefore, the binding of ENL to ESR1 may affect the function of ESR1 in endometriosis. Further Analysis of the harmful effects of PAHs revealed that their metabolites, 1-OHPHE, can bind to GRB2 and interfere with its normal function. GRB2 promotes the recruitment and activation of PI3K, triggering the PI3K-Akt pathway and modulating cell survival and proliferation. Previous studies have shown that GRB2 plays an essential role in the molecular mechanisms underlying endometriosis^64^. The main components of PI3K-Akt pathway are PI3K and Akt/protein kinase B, which, upon activation, inhibit apoptosis and promote cell survival^65^. Continuous activation of PI3K-Akt signaling pathway may lead to chronic inflammation and tissue damage^66,67^. Previous studies have shown that the PI3K/Akt signaling pathway is activated in patients with endometriosis, accompanied by an increase in pyroptosis and inflammatory cytokine levels^68^. Additionally, small molecule inhibitors of the PI3K-Akt pathway have demonstrated therapeutic efficacy in mouse models^69^. Some research shows that PI3K-Akt inhibitor reduced UA by inhibiting the expression of uric acid metabolizing proteins, reducing inflammation, oxidative stress, and mitochondrial apoptosis^70,71^. Studies have shown that the expression of XDH can activate several immune-related signaling pathways, including the PI3K-AKT pathway, thereby inducing cytotoxic immune responses^72^. Meanwhile, one study showed that chemical induced increased ferritin levels, which was mediated by the Akt pathway^73^. Other research has shown that the degradation of Fer (called ferritinophagy) is closely related to the PI3K-AKT pathway. Therefore, exposure to ENL And 1-OHPHE may alter UA and Fer levels, respectively, by interfering with the PI3K-Akt signaling pathway, potentially modulating the risk of endometriosis. The mechanism described in this study is illustrated in Fig. 7.

This study offers several key strengths. Firstly, it utilized a large, nationally representative sample, enhancing both the external validity And statistical power of the findings. Additionally, the study controlled for a range of potential confounders, which helps to reduce bias And confounding in the Analysis. Furthermore, various stratified and sensitivity analyses and distinct statistical approaches, such as WQS and quantile g-computation, were employed to evaluate the combined effects of EDC mixtures on the risk of endometriosis, providing complementary insights and reinforcing the robustness of the results. Additionally, we identified the mediating roles of UA and Fer in the relationships between ENL and 1-OHPHE exposure and the risk of endometriosis, which contributes to a more comprehensive understanding of the harmful effects of EDCs. The other strength of the study was the use of network pharmacology analysis to explore the potential mechanisms underlying the effects of EDCs. Moreover, we found that ENL supplementation could be a promising strategy to mitigate the adverse impacts of PAH exposure on endometriosis risk.

However, the study has some Limitations. First, as a cross-sectional design, it is not possible to draw causal conclusions about the relationship between EDCs and the risk of endometriosis. Future prospective cohort studies with longitudinal follow-up would be valuable to establish temporal relationships and strengthen causal inference between EDC exposure And endometriosis development. Second, the use of single-point urinary samples from NHANES makes it difficult to accurately assess long-term exposure to EDCs, and 24-hour urine samples would likely provide more reliable results. Future studies should consider implementing repeated biomonitoring over multiple time points or using alternative biomarkers with longer half-lives to better capture chronic exposure patterns. Additionally, incorporating personal exposure monitoring devices could provide more comprehensive exposure assessments. However, although the risk of misclassification may still exist, it is most likely non-differential and would therefore primarily attenuate the observed exposure–effect associations^74^. Third, due to the lack of information on exposure to lignans, we were unable to further investigate the role of gut microbiota in influencing the effects of phytoestrogens on endometriosis through the metabolic relationship between gut microbiota, lignans, and ENL. Future research should integrate microbiome analysis with phytoestrogen metabolite profiling to elucidate the complex interplay between gut microbiota, dietary phytoestrogens, and endometriosis risk. Fourth, the study lacks histological or surgical confirmation of endometriosis cases, relying instead on self-reported diagnosis from NHANES questionnaires. This may introduce misclassification bias, as gold standard endometriosis diagnosis requires laparoscopic confirmation. Our study employed various statistical methods and mechanistic analyses as complementary approaches to the observational data in order to improve the reliability of the results. Future research should incorporate clinically validated endometriosis diagnoses to strengthen the reliability of environmental exposure associations.

## Conclusion

This study found that higher ENL levels were associated with a decreased risk of endometriosis, while 1-OHPHE increased the risk under mixed exposure, with both effects mediated by the inflammatory mediators UA And Fer, respectively. ENL influences endometriosis by binding to XDH, thereby inhibiting UA production. Interestingly, elevated ENL levels were shown to mitigate the harmful impact of 1-OHPHE on endometriosis, potentially through a healthy gut microbiota. These findings highlight the critical role of environmental exposures and microbiome interactions in modulating endometriosis risk.

## Supplementary Information

Below is the link to the electronic supplementary material.

Supplementary Material 1

## Data Availability

This study utilizes datasets from the NHANES repository, which can be found at https://www.cdc.gov/nchs/nhanes/. All data is publicly available.
