# Supplementary material for: The effect of phytoestrogens and PAHs on endometriosis and the involvement of gut microbiota, inflammation, and molecular targets
Source: Sci Rep. 2025 Oct 15;15:36027. doi: 10.1038/s41598-025-20042-5 (PMC12528715; doi:10.1038/s41598-025-20042-5)
Supplement: Supplementary file 1 — Supplementary Material 1 [file 41598_2025_20042_MOESM1_ESM.docx]

**Supplementary Text 1** Measurements of chemicals exposure

S1.1. PAEs

PAEs were quantified using enzymatic deconjugation followed by high-performance liquid chromatography coupled with electrospray ionization-tandem mass spectrometry (HPLC-ESI-MS/MS). During the 2001–2002 NHANES cycle, reversed-phase HPLC and atmospheric pressure chemical ionization-tandem mass spectrometry (APCI-MS/MS) were used, whereas from 2003 to 2006, HPLC-ESI-MS/MS was employed for the detection of additional phthalate metabolites. This approach enabled the identification of up to fifteen monoester metabolites of common phthalate diesters in urine, with detection limits in the low parts-per-billion (ppb) range. In cases where the detection result was below the limit of detection, the value for that variable is the detection limit divided by the square root of two ^1^.

S1.2. PAHs

Urinary PAHs were analyzed following enzymatic hydrolysis of urine samples to cleave PAH conjugates. The samples were then subjected to solid-phase extraction, derivatization, and analysis by capillary gas chromatography coupled with high-resolution mass spectrometry (GC/HRMS). The method employed isotope dilution using 13C-labeled internal standards to ensure precise quantification, providing valuable insights into human exposure to PAHs. In cases where the detection result was below the limit of detection, the value for that variable is the detection limit divided by the square root of two ^1^.

S1.3. Phytoestrogens

The assessment of urinary phytoestrogens was conducted using HPLC-MS/MS with enzymatic deconjugation and solid-phase extraction from 2001 to 2002. For the 2003–2004 cycles, HPLC-MS/MS was continued, transitioning to high-performance liquid chromatography coupled with atmospheric pressure photoionization-tandem mass spectrometry (HPLC-APPI-MS/MS) in 2005–2006. These methods were optimized for high precision and sensitivity, with stable-isotope labeled internal standards further enhancing analytical accuracy. In cases where the detection result was below the limit of detection, the value for that variable is the detection limit divided by the square root of two ^1^.

All urine samples were processed, stored, and forwarded to the Division of Environmental Health Laboratory Sciences at the National Center for Environmental Health, CDC for analysis. Specimen collection and processing adhered to the procedures outlined in the NHANES Laboratory/Medical Technologists Procedures Manual (LPM), ensuring stringent quality control and reliable laboratory results.

**Supplementary Table 1** Chemicals in NHANES 2001-2006

| Classification | Chemical | Abbreviation | Detection rate | Median [IQR] |
| --- | --- | --- | --- | --- |
| PAEs | Mono-(2-ethyl)-hexyl phthalate (ng/ml) | MEHP | 73.94% | 2.70[0.85,7.50] |
|  | Mono-(2-ethyl-5-hydroxyhexyl) phthalate (ng/ml) | MEHHP | 98.92% | 18.300[7.40,43.72] |
|  | Mono-(2-ethyl-5-oxohexyl) phthalate (ng/ml) | MEOHP | 97.77% | 12.500[4.80,29.14] |
|  | Mono-(3-carboxypropyl) phthalate (ng/ml) | MCPP | 94.26% | 2.10[1.00,4.40] |
|  | Mono-benzyl phthalate (ng/ml) | MBzP | 98.92% | 9.37[3.60,20.24] |
|  | Mono-ethyl phthalate (ng/ml) | MEP | 99.86% | 139.39[51.18,328.45] |
|  | Mono-isobutyl phthalate (ng/ml) | MiBP | 88.87% | 3.986[1.50,8.78] |
|  | Mono-n-butyl phthalate (ng/ml) | MBP | 99.14% | 21.50[9.40,41.78] |
|  | Mono-n-methyl phthalate (ng/ml) | MMP | 80.38% | 0.90[0.70,2.70] |
| PAHs | 1-Hydroxynaphthalene (ng/l) | 1-OHNAP | 99.93% | 1831.37[743.90,8555.00] |
|  | 1-Hydroxyphenanthrene (ng/l) | 1-OHPHE | 99.56% | 150.45[77.23,290.04] |
|  | 1-Hydroxypyrene (ng/l) | 1-OHPYR | 99.41% | 67.70[32.10,155.97] |
|  | 2-Hydroxyfluorene (ng/l) | 2-OHFLU | 99.85% | 257.18[121.81,784.79] |
|  | 2-Hydroxynaphthalene (ng/l) | 2-OHNAP | 99.93% | 3316.89[1246.56,9300.03] |
|  | 2-Hydroxyphenanthrene (ng/l) | 2-OHPHE | 99.54% | 55.86[27.16,118.42] |
|  | 3-Hydroxyfluorene (ng/l) | 3-OHFLU | 99.33% | 95.09[42.33,384.07] |
|  | 3-Hydroxyphenanthrene (ng/l) | 3-OHPHE | 99.65% | 93.01[45.56,196.15] |
| Phytoestrogens | Daidzein (ng/ml) | Daidzein | 97.20% | 51.512[16.30,175.04] |
|  | Enterodiol (ng/ml) | END | 98.35% | 41.40[14.00,111.00] |
|  | Enterolactone (ng/ml) | ENL | 99.50% | 370.14[100.30,846.86] |
|  | Equol (ng/ml) | EQU | 88.51% | 7.40[2.33,17.10] |
|  | Genistein (ng/ml) | GNS | 99.14% | 22.57[8.90,83.01] |
|  | o-Desmethylangolensin (ng/ml) | O-DMA | 97.35% | 3.70[0.60,23.34] |
| Inflammatory biomarkers | C-reactive protein (mg/dl) | CRP | 100% | 0.24 [0.08, 0.55] |
|  | Ferritin (ug/l) | Fer | 100% | 41.00 [21.00, 74.00] |
|  | Uric acid (mg/dl) | UA | 100% | 4.40 [3.80, 5.10] |
| Lipid metabolism biomarkers | HDL-cholesterol (mg/dL) | HDL-C | 100% | 57.00 [47.00, 67.00] |
|  | LDL-cholesterol (mg/dl) | LDL-C | 100% | 112.00 [92.00, 136.00] |
|  | Total cholesterol (mg/dl) | TC | 100% | 196.00 [173.00, 223.00] |
|  | Triglycerides (mg/dl) | TG | 100% | 101.00 [71.00, 145.00] |

**Supplementary Table 2** Associations between EDCs and endometriosis in NHANES 2001-2006

| Classfication | Exogenous Chemicals | Logistic regression | | PLS-DA |
| --- | --- | --- | --- | --- |
|  |  | *P* | OR (95CI%) | VIP |
| PAEs | MBP | 0.057 | 1.398(0.989,1.975) | 0.739 |
|  | MBzP | 0.006 | 1.354(1.096,1.673) | 0.782 |
|  | MCPP | 0.271 | 1.185(0.872,1.610) | 0.849 |
|  | MEHHP | 0.215 | 0.844(0.643,1.108) | 0.587 |
|  | MEHP | 0.123 | 0.762(0.538,1.079) | 1.011 |
|  | MEOHP | 0.264 | 0.854(0.645,1.131) | 0.639 |
|  | MEP | 0.936 | 1.007(0.839,1.210) | 0.610 |
|  | MiBP | 0.649 | 1.059(0.823,1.363) | 0.327 |
|  | MMP | 0.693 | 1.038(0.859,1.254) | 0.375 |
| PAHs | 1-OHNAP | 0.003 | 1.238(1.082,1.418) | 1.359 |
|  | 1-OHPHE | 0.008 | 1.679(1.153,2.446) | 1.256 |
|  | 1-OHPYR | 0.054 | 1.315(0.995,1.738) | 1.348 |
|  | 2-OHFLU | 0.033 | 1.407(1.029,1.924) | 1.598 |
|  | 2-OHNAP | 0.022 | 1.409(1.053,1.884) | 1.208 |
|  | 2-OHPHE | 0.006 | 1.529(1.133,2.062) | 1.236 |
|  | 3-OHFLU | 0.021 | 1.352(1.050,1.742) | 1.595 |
|  | 3-OHPHE | 0.026 | 1.426(1.045,1.944) | 1.494 |
| Phytoestrogens | Daidzein | 0.989 | 0.999(0.842,1.184) | 0.795 |
|  | END | 0.083 | 0.840(0.688,1.024) | 1.311 |
|  | ENL | 0.040 | 0.828(0.691,0.991) | 1.399 |
|  | EQU | 0.885 | 0.982(0.766,1.260) | 0.265 |
|  | GNS | 0.529 | 1.061(0.879,1.280) | 1.334 |
|  | O-DMA | 0.586 | 0.966(0.849,1.098) | 0.211 |

**Supplementary Table 3** Associations between lipid metabolites and inflammatory biomarkers and endometriosis in NHANES 2001-2006

| Biomarkers | *P* | OR (95%CI) | VIP |
| --- | --- | --- | --- |
| CRP | 0.082 | 1.107(0.987,1.243) | 0.364 |
| Fer | <0.001 | 1.599(1.306,1.957) | 2.108 |
| HDL-C | 0.369 | 0.753(0.402,1.413) | 0.279 |
| LDL-C | 0.574 | 1.249(0.565,2.761) | 0.246 |
| TC | 0.064 | 2.000(0.959,4.170) | 0.515 |
| TG | 0.003 | 1.880(1.251,2.826) | 0.780 |
| UA | 0.048 | 1.803(1.005,3.232) | 1.002 |

**Supplementary Table 4** Associations between EDCs and lipid metabolites and inflammatory biomarkers in NHANES 2001-2006

| Biomarkers | Exogenous Chemicals | Linear regression | | PLS-DA |
| --- | --- | --- | --- | --- |
|  |  | β (95%CI) | *P* | VIP |
| CRP | ***PAEs*** |  |  |  |
|  | MBP | 0.044 (-0.051, 0.139) | 0.357 | 0.331 |
|  | MBzP | 0.145 (0.021, 0.269) | 0.023 | 0.142 |
|  | MCPP | -0.010 (-0.130, 0.109) | 0.863 | 0.230 |
|  | MEHHP | 0.022 (-0.058, 0.102) | 0.583 | 0.214 |
|  | MEHP | -0.012 (-0.084, 0.061) | 0.742 | 0.134 |
|  | MEOHP | 0.042 (-0.036, 0.120) | 0.281 | 0.145 |
|  | MEP | 0.045 (-0.043, 0.133) | 0.306 | 0.347 |
|  | MiBP | 0.085 (-0.029, 0.199) | 0.141 | 0.099 |
|  | MMP | -0.099 (-0.193, -0.004) | 0.041 | 0.635 |
|  | ***PAHs*** |  |  |  |
|  | 1-OHNAP | -0.036 (-0.125, 0.053) | 0.417 | 0.468 |
|  | 1-OHPHE | 0.123 (-0.007, 0.253) | 0.064 | 0.387 |
|  | 1-OHPYR | 0.063 (-0.037, 0.163) | 0.212 | 0.175 |
|  | 2-OHFLU | -0.003 (-0.096, 0.089) | 0.940 | 0.328 |
|  | 2-OHNAP | -0.003 (-0.118, 0.111) | 0.952 | 0.041 |
|  | 2-OHPHE | 0.260 (0.175, 0.344) | <0.001 | 1.152 |
|  | 3-OHFLU | -0.037 (-0.109, 0.036) | 0.316 | 0.574 |
|  | 3-OHPHE | -0.069 (-0.183, 0.046) | 0.233 | 0.527 |
|  | ***Phytoestrogens*** |  |  |  |
|  | Daidzein | -0.082 (-0.150, -0.015) | 0.017 | 0.758 |
|  | END | -0.061 (-0.124, 0.003) | 0.060 | 0.925 |
|  | ENL | -0.154 (-0.209, -0.099) | <0.001 | 1.188 |
|  | EQU | -0.076 (-0.145, -0.006) | 0.033 | 0.633 |
|  | GNS | -0.105 (-0.176, -0.034) | 0.005 | 0.610 |
|  | O-DMA | -0.069 (-0.118, -0.019) | 0.008 | 0.915 |
| Fer | ***PAEs*** |  |  |  |
|  | MBP | -0.001 (-0.087, 0.085) | 0.983 | 0.024 |
|  | MBzP | -0.028 (-0.112, 0.057) | 0.513 | 0.079 |
|  | MCPP | -0.068 (-0.157, 0.020) | 0.128 | 0.333 |
|  | MEHHP | -0.036 (-0.102, 0.030) | 0.272 | 0.578 |
|  | MEHP | -0.071 (-0.131, -0.011) | 0.021 | 1.221 |
|  | MEOHP | -0.051 (-0.125, 0.023) | 0.170 | 0.863 |
|  | MEP | 0.043 (-0.031, 0.118) | 0.249 | 0.159 |
|  | MiBP | -0.042 (-0.143, 0.058) | 0.401 | 0.412 |
|  | MMP | 0.013 (-0.065, 0.091) | 0.732 | 0.247 |
|  | ***PAHs*** |  |  |  |
|  | 1-OHNAP | 0.060 (-0.001, 0.121) | 0.053 | 1.187 |
|  | 1-OHPHE | 0.134 (0.019, 0.248) | 0.023 | 1.187 |
|  | 1-OHPYR | 0.033 (-0.052, 0.118) | 0.444 | 1.028 |
|  | 2-OHFLU | 0.074 (-0.008, 0.156) | 0.076 | 1.396 |
|  | 2-OHNAP | 0.057 (-0.015, 0.129) | 0.117 | 1.005 |
|  | 2-OHPHE | 0.071 (-0.011, 0.153) | 0.086 | 0.860 |
|  | 3-OHFLU | 0.060 (-0.012, 0.132) | 0.101 | 1.486 |
|  | 3-OHPHE | 0.052 (-0.043, 0.148) | 0.276 | 1.158 |
|  | ***Phytoestrogens*** |  |  |  |
|  | Daidzein | -0.029 (-0.074, 0.015) | 0.191 | 0.374 |
|  | END | -0.029 (-0.080, 0.021) | 0.249 | 0.310 |
|  | ENL | -0.023 (-0.070, 0.024) | 0.330 | 0.703 |
|  | EQU | 0.001 (-0.069, 0.071) | 0.984 | 0.335 |
|  | GNS | -0.051 (-0.093, -0.008) | 0.022 | 0.599 |
|  | O-DMA | 0.006 (-0.028, 0.040) | 0.724 | 0.943 |
| HDL-C | ***PAEs*** |  |  |  |
|  | MBP | -0.011 (-0.043, 0.022) | 0.517 | 0.203 |
|  | MBzP | -0.008 (-0.038, 0.022) | 0.610 | 0.152 |
|  | MCPP | 0.006 (-0.024, 0.036) | 0.680 | 0.272 |
|  | MEHHP | 0.012 (-0.014, 0.038) | 0.352 | 0.456 |
|  | MEHP | 0.013 (-0.008, 0.033) | 0.215 | 0.624 |
|  | MEOHP | 0.019 (-0.006, 0.044) | 0.124 | 0.664 |
|  | MEP | 0.008 (-0.006, 0.023) | 0.254 | 0.303 |
|  | MiBP | 0.016 (-0.010, 0.042) | 0.221 | 0.857 |
|  | MMP | 0.035 (0.018, 0.053) | <0.001 | 1.067 |
|  | ***PAHs*** |  |  |  |
|  | 1-OHNAP | -0.015 (-0.031, 0.001) | 0.063 | 0.759 |
|  | 1-OHPHE | -0.013 (-0.044, 0.018) | 0.402 | 0.786 |
|  | 1-OHPYR | -0.019 (-0.045, 0.007) | 0.141 | 0.980 |
|  | 2-OHFLU | -0.041 (-0.060, -0.022) | <0.001 | 1.128 |
|  | 2-OHNAP | -0.039 (-0.056, -0.022) | <0.001 | 1.046 |
|  | 2-OHPHE | -0.030 (-0.056, -0.004) | 0.023 | 0.781 |
|  | 3-OHFLU | -0.038 (-0.054, -0.021) | <0.001 | 1.171 |
|  | 3-OHPHE | -0.035 (-0.066, -0.005) | 0.025 | 0.875 |
|  | ***Phytoestrogens*** |  |  |  |
|  | Daidzein | 0.015 (-0.002, 0.032) | 0.080 | 0.888 |
|  | END | 0.027 (0.009, 0.045) | 0.004 | 1.244 |
|  | ENL | 0.031 (0.021, 0.042) | <0.001 | 1.155 |
|  | EQU | 0.005 (-0.009, 0.020) | 0.488 | 0.798 |
|  | GNS | 0.010 (-0.007, 0.026) | 0.253 | 0.704 |
|  | O-DMA | 0.013 (0.004, 0.022) | 0.006 | 0.937 |
| LDL-C | ***PAEs*** |  |  |  |
|  | MBP | 0.001 (-0.050, 0.051) | 0.970 | 0.794 |
|  | MBzP | -0.005 (-0.035, 0.026) | 0.744 | 1.351 |
|  | MCPP | -0.035 (-0.085, 0.016) | 0.170 | 0.631 |
|  | MEHHP | -0.023 (-0.062, 0.016) | 0.234 | 0.949 |
|  | MEHP | -0.006 (-0.040, 0.029) | 0.745 | 0.871 |
|  | MEOHP | -0.029 (-0.069, 0.011) | 0.151 | 1 |
|  | MEP | -0.024 (-0.055, 0.008) | 0.133 | 1.214 |
|  | MiBP | -0.039 (-0.083, 0.005) | 0.080 | 1.942 |
|  | MMP | -0.007 (-0.036, 0.023) | 0.655 | 0.747 |
|  | ***PAHs*** |  |  |  |
|  | 1-OHNAP | 0.001 (-0.028, 0.030) | 0.963 | 0.737 |
|  | 1-OHPHE | 0.023 (-0.027, 0.074) | 0.361 | 0.664 |
|  | 1-OHPYR | 0 (-0.034, 0.035) | 0.983 | 0.420 |
|  | 2-OHFLU | 0.018 (-0.014, 0.049) | 0.26 | 0.580 |
|  | 2-OHNAP | 0 (-0.033, 0.032) | 0.988 | 0.594 |
|  | 2-OHPHE | 0.025 (-0.014, 0.063) | 0.202 | 0.575 |
|  | 3-OHFLU | 0.019 (-0.007, 0.045) | 0.149 | 0.550 |
|  | 3-OHPHE | 0.029 (-0.017, 0.075) | 0.213 | 0.459 |
|  | ***Phytoestrogens*** |  |  |  |
|  | Daidzein | 0 (-0.024, 0.023) | 0.979 | 0.923 |
|  | END | 0.002 (-0.020, 0.024) | 0.857 | 0.409 |
|  | ENL | -0.005 (-0.025, 0.014) | 0.571 | 0.786 |
|  | EQU | 0.026 (0.002, 0.050) | 0.033 | 1.063 |
|  | GNS | 0.013 (-0.010, 0.036) | 0.258 | 0.982 |
|  | O-DMA | 0.002 (-0.012, 0.017) | 0.758 | 0.910 |
| TC | ***PAEs*** |  |  |  |
|  | MBP | 0.012 (-0.010, 0.034) | 0.288 | 0.433 |
|  | MBzP | -0.003 (-0.019, 0.012) | 0.676 | 0.250 |
|  | MCPP | -0.003 (-0.026, 0.019) | 0.763 | 0.240 |
|  | MEHHP | 0 (-0.018, 0.019) | 0.969 | 0.222 |
|  | MEHP | -0.003 (-0.021, 0.015) | 0.731 | 0.390 |
|  | MEOHP | 0 (-0.019, 0.019) | 0.980 | 0.284 |
|  | MEP | 0.004 (-0.010, 0.018) | 0.566 | 0.286 |
|  | MiBP | -0.005 (-0.027, 0.017) | 0.630 | 0.933 |
|  | MMP | 0.013 (-0.004, 0.030) | 0.122 | 0.848 |
|  | ***PAHs*** |  |  |  |
|  | 1-OHNAP | -0.008 (-0.019, 0.003) | 0.166 | 0.714 |
|  | 1-OHPHE | 0.017 (-0.008, 0.041) | 0.185 | 1.067 |
|  | 1-OHPYR | 0.004 (-0.011, 0.019) | 0.597 | 0.578 |
|  | 2-OHFLU | 0 (-0.014, 0.015) | 0.965 | 0.671 |
|  | 2-OHNAP | 0.002 (-0.012, 0.016) | 0.783 | 0.720 |
|  | 2-OHPHE | 0.012 (-0.006, 0.030) | 0.199 | 1.117 |
|  | 3-OHFLU | -0.003 (-0.015, 0.010) | 0.670 | 0.858 |
|  | 3-OHPHE | 0.008 (-0.015, 0.031) | 0.477 | 0.311 |
|  | ***Phytoestrogens*** |  |  |  |
|  | Daidzein | -0.002 (-0.012, 0.009) | 0.762 | 1.342 |
|  | END | 0.008 (-0.001, 0.018) | 0.093 | 0.817 |
|  | ENL | -0.003 (-0.012, 0.007) | 0.532 | 0.822 |
|  | EQU | 0.015 (0.004, 0.027) | 0.011 | 1.607 |
|  | GNS | 0.004 (-0.006, 0.014) | 0.448 | 1.292 |
|  | O-DMA | 0.003 (-0.003, 0.009) | 0.386 | 1.310 |
| TG | ***PAEs*** |  |  |  |
|  | MBP | 0.047 (-0.012, 0.106) | 0.113 | 0.451 |
|  | MBzP | 0.034 (-0.028, 0.096) | 0.277 | 0.165 |
|  | MCPP | -0.008 (-0.08, 0.064) | 0.830 | 0.114 |
|  | MEHHP | 0.013 (-0.064, 0.089) | 0.738 | 0.114 |
|  | MEHP | -0.009 (-0.085, 0.068) | 0.819 | 0.594 |
|  | MEOHP | 0.003 (-0.071, 0.076) | 0.943 | 0.062 |
|  | MEP | 0.026 (-0.036, 0.087) | 0.404 | 0.316 |
|  | MiBP | 0.055 (-0.011, 0.122) | 0.101 | 0.504 |
|  | MMP | -0.012 (-0.092, 0.067) | 0.754 | 0.791 |
|  | ***PAHs*** |  |  |  |
|  | 1-OHNAP | 0.008 (-0.038, 0.053) | 0.743 | 0.490 |
|  | 1-OHPHE | 0.112 (0.023, 0.202) | 0.015 | 1.341 |
|  | 1-OHPYR | 0.068 (0.015, 0.120) | 0.013 | 1.107 |
|  | 2-OHFLU | 0.041 (-0.008, 0.090) | 0.096 | 0.856 |
|  | 2-OHNAP | 0.065 (0.024, 0.106) | 0.003 | 0.808 |
|  | 2-OHPHE | 0.134 (0.079, 0.189) | <0.001 | 1.837 |
|  | 3-OHFLU | 0.016 (-0.024, 0.057) | 0.418 | 1.008 |
|  | 3-OHPHE | 0.042 (-0.031, 0.115) | 0.254 | 1.011 |
|  | ***Phytoestrogens*** |  |  |  |
|  | Daidzein | -0.031 (-0.063, 0.001) | 0.057 | 0.308 |
|  | END | -0.027 (-0.059, 0.006) | 0.112 | 0.600 |
|  | ENL | -0.062 (-0.094, -0.031) | <0.001 | 1.332 |
|  | EQU | 0.044 (-0.002, 0.091) | 0.060 | 1.268 |
|  | GNS | -0.008 (-0.047, 0.031) | 0.687 | 0.644 |
|  | O-DMA | -0.030 (-0.053, -0.006) | 0.015 | 0.759 |
| UA | ***PAEs*** |  |  |  |
|  | MBP | 0 (-0.022, 0.022) | 0.990 | 0.284 |
|  | MBzP | -0.011 (-0.033, 0.011) | 0.319 | 0.222 |
|  | MCPP | -0.017 (-0.035, 0.002) | 0.072 | 0.278 |
|  | MEHHP | -0.002 (-0.020, 0.016) | 0.820 | 0.471 |
|  | MEHP | -0.023 (-0.040, -0.006) | 0.009 | 0.940 |
|  | MEOHP | -0.003 (-0.022, 0.016) | 0.749 | 0.499 |
|  | MEP | 0.005 (-0.011, 0.021) | 0.557 | 0.331 |
|  | MiBP | -0.001 (-0.022, 0.019) | 0.903 | 0.361 |
|  | MMP | -0.019 (-0.037, -0.001) | 0.038 | 0.712 |
|  | ***PAHs*** |  |  |  |
|  | 1-OHNAP | -0.013 (-0.023, -0.003) | 0.012 | 0.178 |
|  | 1-OHPHE | 0.001 (-0.030, 0.032) | 0.949 | 0.514 |
|  | 1-OHPYR | -0.014 (-0.035, 0.006) | 0.171 | 0.632 |
|  | 2-OHFLU | -0.013 (-0.029, 0.004) | 0.126 | 0.478 |
|  | 2-OHNAP | -0.005 (-0.023, 0.012) | 0.535 | 0.375 |
|  | 2-OHPHE | 0.020 (-0.005, 0.045) | 0.121 | 0.529 |
|  | 3-OHFLU | -0.011 (-0.027, 0.004) | 0.157 | 0.423 |
|  | 3-OHPHE | -0.006 (-0.029, 0.018) | 0.631 | 0.125 |
|  | ***Phytoestrogens*** |  |  |  |
|  | Daidzein | -0.003 (-0.017, 0.010) | 0.610 | 0.629 |
|  | END | -0.012 (-0.026, 0.001) | 0.076 | 1.339 |
|  | ENL | -0.021 (-0.033, -0.010) | <0.001 | 1.740 |
|  | EQU | -0.009 (-0.027, 0.008) | 0.271 | 0.878 |
|  | GNS | 0.002 (-0.010, 0.015) | 0.706 | 0.559 |
|  | O-DMA | 0.003 (-0.006, 0.011) | 0.520 | 0.703 |

**Supplementary Table 5** Subgroup analysis of the association between ENL

| Group | OR (95%CI) | P-value | VIP |
| --- | --- | --- | --- |
| Age |  |  |  |
| Age<35 | 0.691 (0.471, 1.016) | 0.06 | 1.482 |
| Age>=35 | 0.857 (0.713, 1.03) | 0.098 | 1.022 |
| Race |  |  |  |
| Non-Hispanic | 0.843 (0.706, 1.007) | 0.059 | 1.125 |
| Hispanic | 0.652 (0.397, 1.07) | 0.087 | 1.652 |
| BMI |  |  |  |
| Normalweight | 0.96 (0.77, 1.199) | 0.714 | 0.35 |
| Overweight | 0.748 (0.551, 1.014) | 0.06 | 1.739 |
| Obesity | 0.716 (0.54, 0.949) | 0.021 | 1.134 |
| Smoker |  |  |  |
| Never smoker | 0.816 (0.648, 1.028) | 0.083 | 1.161 |
| Former smoker | 0.863 (0.654, 1.139) | 0.288 | 0.467 |
| Current smoker | 0.886 (0.651, 1.207) | 0.43 | 1.604 |
| Alcohol |  |  |  |
| Alcohol No | 0.818 (0.645, 1.038) | 0.096 | 0.682 |
| Alcohol Yes | 0.83 (0.673, 1.024) | 0.081 | 1.385 |
| Education |  |  |  |
| High school and below | 1.011 (0.71, 1.439) | 0.952 | 0.366 |
| High school graduate/GED or equivalent | 0.866 (0.603, 1.243) | 0.423 | 0.804 |
| Some college or AA degree | 0.793 (0.615, 1.023) | 0.073 | 1.441 |
| College graduate or above | 1.007 (0.643, 1.577) | 0.974 | 0.883 |
| PIR |  |  |  |
| PIR ≤1 | 0.858 (0.609, 1.209) | 0.37 | 0.569 |
| PIR 1-3 | 0.863 (0.645, 1.154) | 0.311 | 1.277 |
| PIR >3 | 0.803 (0.625, 1.032) | 0.085 | 1.335 |
| Pregnancy |  |  |  |
| pregNo | 0.831 (0.693, 0.995) | 0.045 | 1.276 |
| pregYes | 0.882 (0.533, 1.458) | 0.614 | 1.118 |
| Age of menarche |  |  |  |
| <13 | 0.851 (0.681, 1.064) | 0.152 | 1.092 |
| >=13 | 0.804 (0.64, 1.011) | 0.061 | 1.336 |
| Number of pregnancies |  |  |  |
| <3 | 0.747 (0.572, 0.976) | 0.033 | 1.96 |
| >=3 | 0.88 (0.722, 1.072) | 0.197 | 0.664 |

**Supplementary Table 6** Subgroup analysis of the association between 1-OHPHE

| Group | OR (95%CI) | P-value | VIP |
| --- | --- | --- | --- |
| Age |  |  |  |
| Age<35 | 2.235 (1.126, 4.433) | 0.022 | 1.355 |
| Age>=35 | 1.487 (0.934, 2.368) | 0.092 | 1.163 |
| Race |  |  |  |
| Non-Hispanic | 1.601 (1.069, 2.398) | 0.023 | 1.054 |
| Hispanic | 2.536 (0.676, 9.508) | 0.158 | 1.08 |
| BMI |  |  |  |
| Normalweight | 1.7 (0.813, 3.556) | 0.154 | 1.359 |
| Overweight | 1.475 (0.867, 2.511) | 0.147 | 0.898 |
| Obesity | 2.126 (0.897, 5.039) | 0.085 | 1.053 |
| Smoker |  |  |  |
| Never smoker | 1.732 (0.709, 4.231) | 0.221 | 0.881 |
| Former smoker | 1.159 (0.418, 3.213) | 0.769 | 0.591 |
| Current smoker | 1.401 (0.754, 2.603) | 0.275 | 1.357 |
| Alcohol |  |  |  |
| Alcohol No | 1.2 (0.643, 2.241) | 0.556 | 1.594 |
| Alcohol Yes | 1.895 (1.244, 2.888) | 0.004 | 1.285 |
| Education |  |  |  |
| High school and below | 4.062 (1.532, 10.769) | 0.006 | 1.323 |
| High school graduate/GED or equivalent | 1.428 (0.76, 2.685) | 0.258 | 1.063 |
| Some college or AA degree | 1.611 (0.824, 3.15) | 0.158 | 0.985 |
| College graduate or above | 1.295 (0.336, 4.989) | 0.698 | 1.266 |
| PIR |  |  |  |
| PIR ≤1 | 2.583 (1.122, 5.946) | 0.027 | 1.273 |
| PIR 1-3 | 1.275 (0.677, 2.403) | 0.443 | 0.731 |
| PIR >3 | 1.711 (0.817, 3.585) | 0.15 | 1.15 |
| Pregnancy |  |  |  |
| pregNo | 1.632 (1.118, 2.382) | 0.012 | 1.226 |
| pregYes | 4.701 (1.762, 12.545) | 0.003 | 1.513 |
| Age of menarche |  |  |  |
| <13 | 1.571 (0.984, 2.508) | 0.058 | 1.089 |
| >=13 | 1.811 (0.955, 3.432) | 0.068 | 1.381 |
| Number of pregnancies |  |  |  |
| <3 | 1.503 (0.681, 3.316) | 0.305 | 0.896 |
| >=3 | 1.868 (1.18, 2.956) | 0.009 | 1.249 |

**Supplementary Table 7** Associations of significant PAHs with the risk of endometriosis stratified by ENL levels in US women

|  | Exogenous Chemicals | Logistic regression | | PLS-DA |
| --- | --- | --- | --- | --- |
|  |  | OR (95%CI) | *P* | VIP |
| Low ENL levels | 1-OHNAP | 1.310 (1.047, 1.640) | 0.019 | 1.327 |
|  | 1-OHPHE | 1.914 (1.217, 3.011) | 0.006 | 1.274 |
|  | 2-OHFLU | 1.452 (1.036, 2.035) | 0.031 | 1.560 |
|  | 2-OHNAP | 1.537 (1.155, 2.046) | 0.004 | 1.215 |
|  | 2-OHPHE | 1.669 (1.147, 2.427) | 0.009 | 1.207 |
|  | 3-OHFLU | 1.370 (1.036, 1.813) | 0.028 | 1.464 |
|  | 3-OHPHE | 1.476 (1.027, 2.120) | 0.036 | 1.431 |
| High ENL levels | 1-OHNAP | 1.170 (0.951, 1.440) | 0.133 | 1.195 |
|  | 1-OHPHE | 1.260 (0.645, 2.462) | 0.490 | 1.086 |
|  | 2-OHFLU | 1.282 (0.745, 2.206) | 0.361 | 1.357 |
|  | 2-OHNAP | 1.230 (0.789, 1.920) | 0.352 | 0.920 |
|  | 2-OHPHE | 1.266 (0.853, 1.881) | 0.235 | 0.861 |
|  | 3-OHFLU | 1.277 (0.847, 1.926) | 0.236 | 1.517 |
|  | 3-OHPHE | 1.243 (0.780, 1.980) | 0.352 | 1.298 |


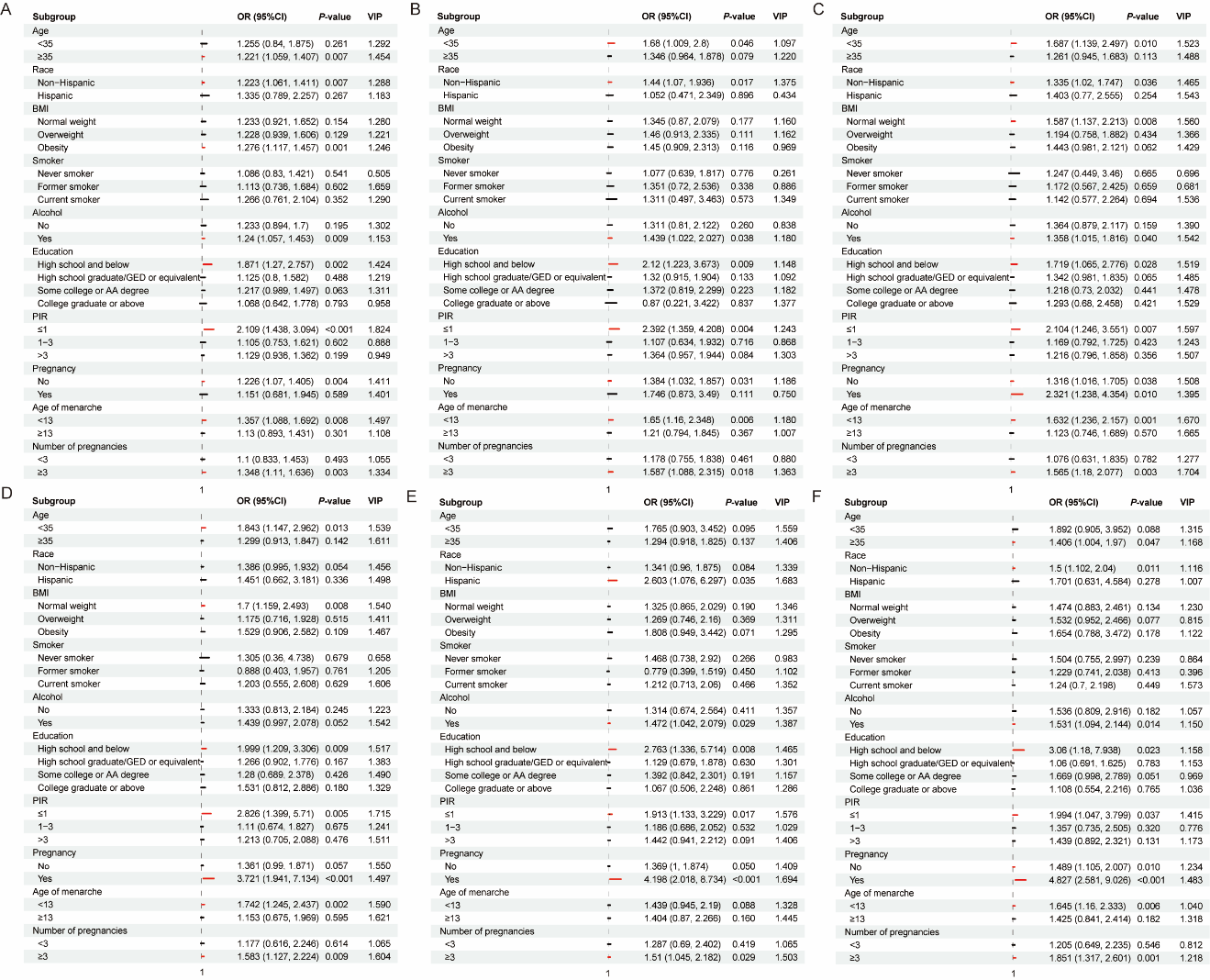
 **Supplementary Figure 1** **Subgroup analysis.** **(A)** 1-OHNAP. **(B)** 2-OHNAP. **(C)** 3-OHFLU. **(D)** 2-OHFLU. **(E)** 3-OHPHE. **(F)** 2-OHPHE.

1 Bingru, L. *et al.* Association between endocrine disrupting chemicals and female infertility: a study based on NHANES database. *Frontiers in public health* **13**, 1608861, doi:10.3389/fpubh.2025.1608861 (2025).
